# On Integral INICS Aromaticity of Pyridodiazepine Constitutional Isomers and Tautomers

**DOI:** 10.3390/molecules28155684

**Published:** 2023-07-27

**Authors:** Małgorzata Jarończyk, Sławomir Ostrowski, Jan Cz. Dobrowolski

**Affiliations:** 1 National Medicines Institute, 30/34 Chełmska Street, 00-725 Warsaw, Poland; 2 Institute of Chemistry and Nuclear Technology, 16 Dorodna Street, 03-195 Warsaw, Poland; s.ostrowski@ichtj.waw.pl

**Keywords:** pyridodiazepine, tautomerism, aromaticity, integral NICS, electron density at RCP

## Abstract

The structure, energetics, and aromaticity of c.a. 100 constitutional isomers and tautomers of pyrido[m,n]diazepines (m = 1, 2; n = 2, 3, 4, 5; m ≠ n) were studied at the B3LYP/cc-pVTZ level. The pyrido[1,3]diazepines appear the most, while pyrido[2,4]diazepines are the least stable (ca. 26 kcal/mol). In the pyrido[1,n]diazepine group (n = 2–5), the [1,5] isomers are higher in energy by ca. 4.5 kcal/mol and the [1,4] ones by ca. 7 kcal/mol, and the pyrido[1,2]diazepines are the least stable (ca. 20 kcal/mol). All the most stable pyrido[1,n]diazepines have N-atoms near the ring’s junction bond but on opposite sites. The most stable [2,n]-forms are also those with the pyridine ring N6-atom near the junction bond. Surprisingly, for the [1,2]-, [1,3]-, and [1,4]-isomer condensation types of pyridine and diazepine rings, the same N9 > N7 > N6 > N8 stability pattern obeys. The stability remains similar in a water medium simulated with the Polarizable Continuum Model of the solvent and is conserved when calculated using the CAM-B3LYP or BHandHlyp functionals. The ring’s aromaticity in the pyridine[m,n]diazepines was established based on the integral INICS index resulting from the NICSzz-scan curves’ integration. The integral INICS index is physically justified through its relation to the ringcurrent as demonstrated by Berger, R.J.F., et al. Phys. Chem. Chem. Phys. 2022, 24, 624. The six-membered pyrido rings have negative INICS_ZZ_ indices and can be aromatic only if they are not protonated at the N-atom. All protonated pyrido and seven-membered rings exhibit meaningful positive INICS_ZZ_ values and can be assigned as antiaromatic. However, some non-protonated pyrido rings also have substantial positive INICS_ZZ_ indices and are antiaromatic. A weak linear correlation (*R*^2^ = 0.72) between the INICS_ZZ_ values of the pyridine I(6) and diazepine I(7) rings exists and is a consequence of the communication between the π-electron systems of the two rings. The juxtaposition of the INICS descriptor of the six- and seven-membered rings and diverse electron density parameters at the Ring Critical Points (RCP) revealed good correlations only with the Electrostatic Potentials from the electrons and nuclei (ESPe and ESPn). The relationships with other RCP parameters like electron density and its Laplacian, total energy, and the Hamiltonian form of kinetic energy density were split into two parts: one nearly constant for the six-membered rings and one linearly correlating for the seven-membered rings. Thus, most of the electron density parameters at the RCP of the six-membered rings of pyridodiazepines practically do not change with the diazepine type and the labile proton position. In contrast, those of the seven-membered rings display aromaticity changes in the antiaromatic diazepine with its ring structural modifications.

## 1. Introduction

Tautomerism, first defined by Laar in 1885 [1], is an isomerism consisting of a shift of an atom/ion or a functional group from one molecule center to another with simultaneous multiple bond(s) rearrangements [2]. Consequently, both the tautomer’s molecular connectivity and charge distribution are altered. In the prototropic tautomerism, a proton is shifted, e.g., [3,4,5,6,7]. Prototropism is essential for many biological and biochemical processes [8]. For example, it is a critical factor in modifying the properties of neutral or protonated bases in nucleic acids, leading to mispairing and, subsequently, mutations [9,10]. Also, tautomerism is responsible for various simple carbohydrate forms in biosystems [11]. Many pharmaceutically active molecules, e.g., metformin [12,13], containing five- or six-membered bi- and multi-heterocyclic ring systems, can exist as two or more prototropic structures [14]. Therefore, studying, understanding, and predicting the possible tautomeric equilibria of heterocycles is crucial, as they are building blocks for molecules important for biology, pharmacy, and medicine [15,16,17,18,19,20,21,22,23].

Aromaticity is one of the key concepts in organic chemistry, facilitating an understanding of the reactivity and properties of cyclic unsaturated compounds [24]. However, it cannot be derived directly from quantum chemistry formalism and requires an enumerative definition [24,25], addressing the (1) energetic [26], (2) geometric [27], (3) magnetic [28], (4) electronic [29], (5) chemical reactivity [26], (6) graph theory [30,31], and other criteria [24]. Any of these criteria allows us to describe the aromaticity quantitatively [24,25,26,27,28,29,30,31]; however, the geometric HOMA-like [5,27,32,33,34] and magnetic NICS-derived indices seem the most popular. Here, we use the newly defined integral NICS (INICS) index. The NICS index was initially established as the opposite value of the absolute magnetic shielding calculated at the ring center (NICS(0)). The ring is aromatic, non-aromatic, and antiaromatic for the index equal to −10.0, 0.0, and 10.0 ppm, respectively [35]. Soon, improved NICS indices filtering the undesirable σ-orbital contribution were introduced: NICS(1) taken at 1 Å over the ring center [36], NICS(π) [37], NICS_ZZ_ [38], and NICS_πZZ_ [39]. For a decade, the NICS scan showing diamagnetic and paramagnetic ring currents has been used [40]. Nevertheless, in 2019, Stanger suggested that the integrated value of NICSπ_ZZ_, ∫ NICSπ_ZZ_, is also worth looking at [41]. Moreover, as Berger et al. showed in 2022, the NICS integral parameter has a physical justification related to the ring current via Ampère-Maxwell’s law [42]. They found that on this basis, the integration scheme combined with the stagnation graph analysis makes it possible to automatically calculate the current in individual rings and all the vortices present in the molecule [42]. A more advanced use of the law was also published in Ref. [43]. Following Stanger’s idea, in 2022, we studied the behavior of the integral NICS index, INICS, to evaluate the aromaticity of aromatic amino acids [44]. We found that INICS is the most robust and indicative index out of several other NICS indices taken at a particular point of the NICS scan, such as 0, 1, and the curve extrema. The calculations of newly defined indices were made possible thanks to the ARONICS program written in our group [45].

Pyridodiazepines are benzodiazepine analogs with an extra N-atom incorporated into the benzene ring, i.e., they are bicyclic systems in which the six-membered pyridine ring is fused with a non-planar seven-membered diazepine one. The former is well-recognized as a therapeutic agent. Pyrrolobenzodiazepines, the antitumor antibiotics, have been studied since the mid-1960s [46,47,48]. Their discovery inspired research on benzodiazepines and benzodiazepinones, now among the widely used psychotropic drugs. Their pharmacological profile covers anxiolytic, sedative–hypnotic, muscle relaxant, and anticonvulsive effects, making them indispensable to current neuropsychiatric therapy [49].

The fantastic therapeutic success of benzodiazepines directed attention to their derivatives or analogs [50]. It has been shown that Lopirazepam, a never-marketed pyridodiazepine analog of Lorazepam, exhibits anxiolytic and hypnotic properties [51,52]. Zapizolam [53], the pyridodiazepine analog of Triazolam, shows benzodiazepine-like sedative and anxiolytic effects. Also, some pyridodiazepines exhibit cytotoxic activity [54,55,56]. Benzodiazepines, pyridodiazepines, and compositions are viable pharmaceuticals for treating neurodegenerative or neuropsychiatric disorders [57,58]. SAR studies suggested that pyridodiazepine amines may be highly selective anti-Helicobacter pylori agents [59]. Also, dipyridodiazepinones were approved to treat HIV infections in humans [60,61].

Pirenzepine, a tricyclic pyridobenzodiazepinone active towards the muscarinic receptor [62], is approved to treat peptic ulcers [63] and is effective at triggering scleral metabolic changes in patients with myopia [64]. Some tricyclic pyridodiazepines exhibit good analgesic and neuroleptic activity as well as inhibiting effects on tumor cells [65]. Several tricyclic diazepines, including A-ring-modified pyridodiazepines fused with a five-membered C-ring, revealed the C-ring role for DNA binding affinity and cytotoxicity [66]. Some tricyclic and tetracyclic derivatives of the benzodiazepine, pyridodiazepine, and pyrimidodiazepine types fused with 1,4-dihydropyridines have a GABAergic and modulating action on calcium channels that can be used to treat cardiovascular, cerebrovascular, neurodegenerative, neuropsychiatric, and neurologic diseases [67].

Pyridodiazepines are heterobicyclic molecules that are promising as building blocks for new medicines. Nevertheless, compared to the benzodiazepine analogs, they garnered less attention in experimental studies. As far as we know, there has been no systematic computational study on their structure, tautomerism, and aromaticity. Here, we demonstrate that the non-planarity of the diazepine ring condensed differently from the pyridine one produces a variety of situations that cannot be foreseen based solely on chemical intuition, which can have important implications for further experimental studies.

The aim of this study was twofold. The first was to gather knowledge about the relationship between pyridodiazepines’ structure, tautomerism, stability, and aromaticity. We expect information on the structure–stability can foster the rational synthesis of potential new therapeutic agents. The second was to test our new integral NICS (INICS) index and the ARONICS program written in our group [45]. The program can find the INICS for non-planar rings, which are present in almost all pyridodiazepines. We found that the most stable pyrido[1,n]diazepines are condensed similarly; the N-atoms are near the ring’s junction bond but on opposite sites. We also found that for the [1,2]-, [1,3]-, and [1,4]-isomer condensation types, the same N9 > N7 > N6 > N8 stability pattern obeys, while for the [1,5]-type, it is simplified to N9(=N6) > N7(=N8) due to symmetry. The comparison of INICS with AIM parameters in RCP demonstrated that only the electrostatic potentials of electrons and nuclei, ESPe and ESPn, provided good correlations and can uniformly reveal aromaticity changes in aromatic and antiaromatic systems. On the other hand, the relationships between INICS and any other parameters in RCP were split into two parts: one for the six- and the other for the seven-membered ring.

## 2. Results and Discussion

**Scheme 1:**
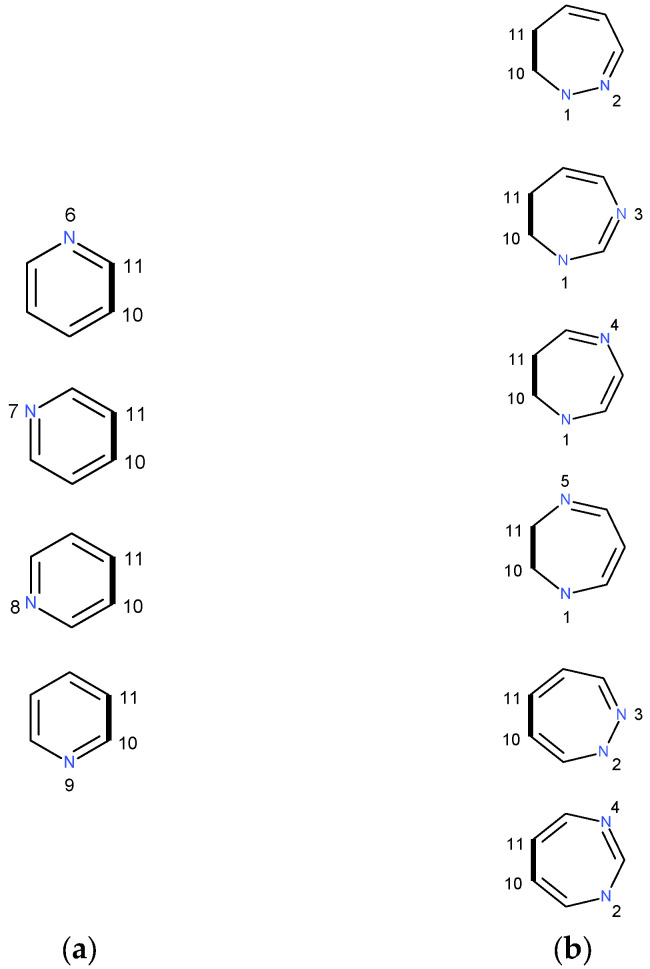
Various condensations of pyridine (**a**) and [n,m]diazepine (**b**) rings through the C10–C11 junction bond to form pyridino[m,n] benzodiazepine. The structures are hydrogen depleted. The condensation causes a redistribution of the electron charge.

**Scheme 2:**
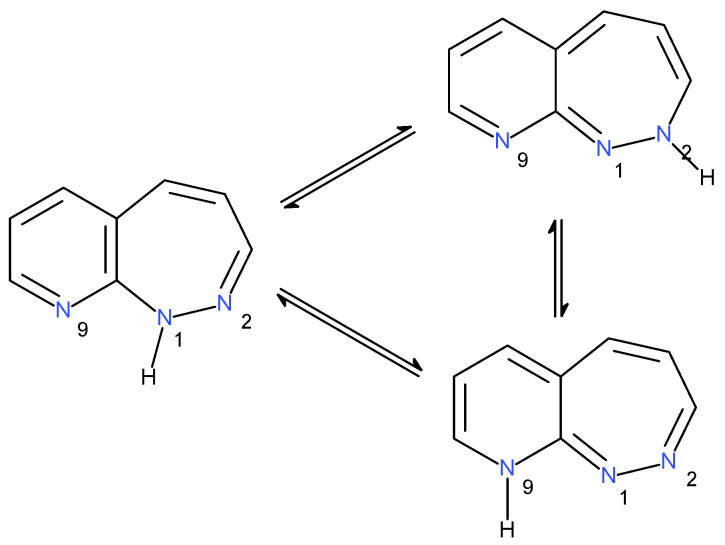
Example of tautomeric equilibria between the N1-H, N2-H, and N9-H tautomers of the pyrido[1,2]diazepines.

The pyridodiazepines considered in this study do not have an N-atom placed in the ring-junction position. The systematic nomenclature of the bicyclic systems with three heteroatoms incorporated into two rings is complex (see Supplementary Materials). Therefore, we introduce a unified atom’s notation and labeling, enabling the easy juxtaposition of data obtained for different tautomers of different pyridodiazepine isomers (Scheme 1). The presence of two pyridine and one pyrimidine type of nitrogen atom in the studied pyridodiazepines makes the coexistence of three tautomers possible (Scheme 2). The seven-membered diazepine ring is usually slightly non-planar and adopts a boat-like or twist-chair conformation [68,69,70,71]. However, for some pyridodiazepines, the planar conformation is more stable than the non-planar one (planarity, Table 1). The sole diazepine rings are known to be non-planar [72,73,74]. Also, in benzodiazepines, the seven-membered rings adopt mainly a twist-chair/distorted-boat conformation [70,75,76]. Surprisingly, some of the studied pyridodiazepines tend to be planar. This makes studies on conformational/tautomeric equilibria more challenging.

### 2.1. Energetics

The pyridodiazepines’ stability (Gibbs free energy differences, ΔG, T = 298.15 K, p = 1 atm) is established at various levels of detail considering types of (i) [m,n]diazepine isomers (DI, ΔG_DI_); (ii) pyridine–diazepine rings’ condensation (PDC, ΔG_PDC_); and (iii) tautomers, (T, ΔG_T_). In the former, structures with different N-atom positions in the diazepine ring are compared (m = 1, n = 2–5; m = 2, n = 3,4); in the following, structures with different N-atom positions in the pyridine ring are compared, while the latter structures with different positions of the labile H-atom are compared. To establish ΔG_DI_, the most stable tautomers of the most stable condensation types are compared, while to establish ΔG_PDC_, the most stable tautomers are compared (Table 1 and Table 2).

#### 2.1.1. [m,n]diazepine Isomers

**Scheme 3:**
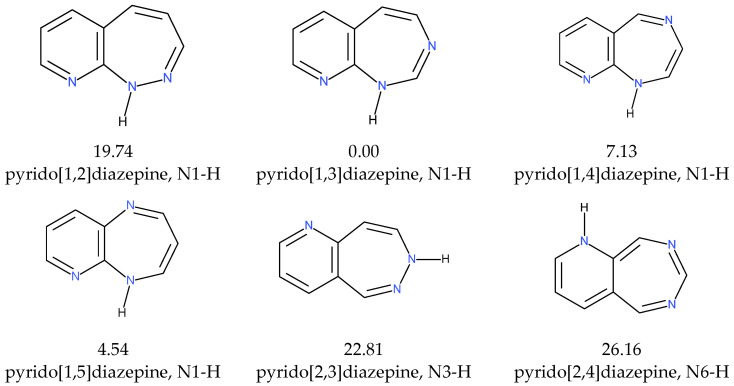
The most stable forms of pyrido[m,n]diazepines for given m and n positions of the N-atoms. The energy differences are given as ΔG_DI_ (kcal/mol).

Among all pyrido[m,n]diazepine isomers, pyrido[1,3]diazepines are the most stable, and pyrido[2,4]diazepines are the least stable, by a matter of ca. 26 kcal/mol. In the pyrido[1,n]diazepine group (n = 2–5), the [1,5] isomers are higher in energy by ca. 4.5 kcal/mol and the [1,4] ones by ca. 7 kcal/mol, and the pyrido[1,2]diazepines are the least stable, by a matter of ca. 20 kcal/mol (Scheme 3).

**Figure 1:**
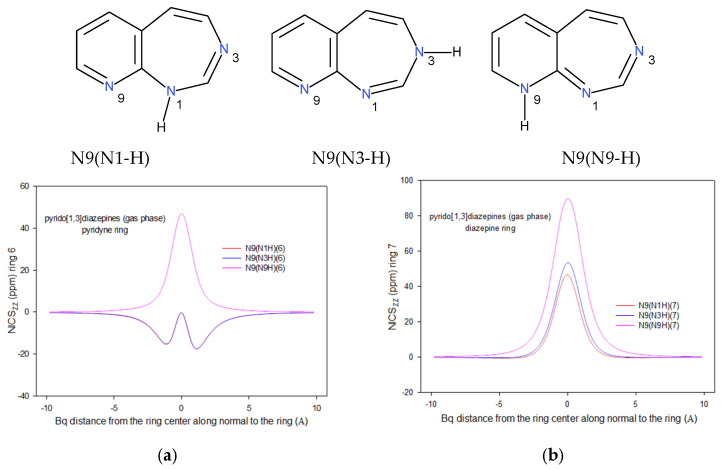
The NICSzz−scans vs. distance from the ring center along normal to the ring for the most stable pirydo[1,3]diazepines for N9 condensation type. Scans (**a**) refer to the pyridine, whereas (**b**) to the [1,3]diazepine ring. Bq stands for the point in which NICSZZ was determined.

Interestingly, all the most stable forms are condensed similarly because the N-atoms are near the ring’s junction bond but on opposite sites (Table 1; Figure 1). In the [1,2], [1,3], and [1,4] isomers, the pyridine N-atom is placed in position 9, and the labile H-atom is attached to the N1-atom, whereas in the case of the [1,5]-isomer, the pyridine N-atom is placed in position 6, and the labile H-atom is attached to the N5-atom (which is, by symmetry, equivalent to the positions N9 and N1-H).

In the group of pyrido[2,n]diazepines (n = 3, 4), [2,3]-isomers are more stable than [2,4] ones by ca. 4 kcal/mol. As before, the most stable [2,n]-forms are those with the pyridine ring N6-atom near the junction bond, but in the case of [2,3]-isomers, the labile H atom is positioned at the N3-atom, whereas in the case of the [2,4] isomers, it is unexpectedly located at the same pyridine N6 atom.

#### 2.1.2. Pyridine–Diazepine Rings Condensation

**Scheme 4:**
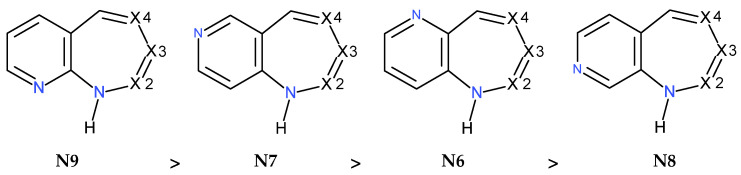
The stability of pyridine–diazepine condensation in the pyrido[1,n]diazepine isomers (n = 2, 3, 4) decreases from left to right. For given n, in the most stable isomers, the labile H-atom is always positioned at the N1-atom. One of the X1, X2, and X3-atoms is N, while the others are C. See text for [1,5]- and [2,n]-isomers.

Now, for each pyrido[1,n]diazepine isomer (n = 2, 3, 4, 5), the DI type, the energetics of the pyridine–diazepine rings’ condensation, the PDC type is compared, remembering that for each pyrido[1,n]diazepine, the N9 condensation provides the most stable structure (Table 1). Interestingly, for the [1,2]-, [1,3]-, and [1,4]-isomers, the same stability pattern obeys (Scheme 4): the second stable is N7, which is more stable than N6, which is more stable than N8: N9 > N7 > N6 > N8 and the energy differences are, respectively, as follows: 0.0, 4.2, 4.9, 6.2 kcal/mol for the [1,2] = isomers; 0.0, 3.6, 3.9, 4.8 kcal/mol, for the [1,3] = isomers; and 0.0, 5.0; 6.6, 7.5 kcal/mol, for the [1,4]-isomers. In the [1,5]-isomers, the N1 and N5 and N6 and N9 positions are symmetrically equivalent; therefore, both the N9 and N6 condensations are the most stable, while N7 and N8 are less stable by 5.9 kcal/mol.

Two pyrido[2,n]diazepine isomers (n = 3, 4) must be considered individually. For [2,3]-isomers, the condensation stability sequence is as follows: N6 > N7 > N9 > N8 with the following Gibbs free energy differences: 0.0, 1.8, 17.9, and 18.2 kcal/mol, respectively (Table 2). In the case of [2,4]-isomers, the N7 and N8, as well as N6 and N9 positions, are symmetrically equivalent; therefore, both N7 and N8 condensations are the most stable, and both condensations N6 and N9 are less stable by 26.2 kcal/mol.

#### 2.1.3. Tautomers

Now, let us look at the tautomeric equilibria within a given diazepine ring type and condensation. In the case of [1,2]-isomers, the N1-H tautomer is the most stable for every pyridine condensation type, and the other tautomers are less stable by at least 18 kcal/mol. The N2-H is the second stable, except for the N9 type of condensation, for which the energy of the N9-H tautomer is ca. 21 kcal/mol and is lower than that of the N2-H one by ca. 4.5 kcal/mol.

In the case of [1,3]-isomers, for every pyridine condensation type but N9, the N3-H tautomer is the most stable, but the N1-H is only slightly energetically higher, a matter of 2.1, 0.5, and 1.1 kcal/mol for N6, N7, and N8 condensation types, respectively. For the N9 condensed structures, the N1-H tautomer is significantly more stable than N3-H by ca. 6.1 kcal/mol. The N9-H tautomer is always the least stable by at least 12 kcal/mol.

For all [1,4]-isomers, the N1-H tautomer is the most stable. Among the other two tautomers, N8-H is always more stable than N4-H but less stable than N1-H by at least 13 kcal/mol.

In the case of [1,5]-isomers, we have only two condensation forms, N9 and N8 (or N6 and N7), and for N9, the N1-H tautomer is the most stable; then, N5-H (8.1 kcal/mol) and N9-H (11.6 kcal/mol) finally, whereas for N8, the N5-H tautomer is the most stable, then N1-H (0.6 kcal/mol) and finally N8-H (13.2 kcal/mol).

Interestingly, for [2,3]-isomers, for the N6 and N7 condensation types, the N3-H tautomers are the most stable, whereas, for the N8 and N9 condensation types, the N2-H tautomers are the most stable. However, in the former case, the other two tautomers are quite unstable (less stable by at least 18 kcal/mol); in the latter case, the N2-H tautomer is less stable by 1.9 (N8) and 0.8 (N9) kcal/mol, whereas the other one is less stable by 4.6 (N8) and 11.4 (N9) kcal/mol.

The [2,4]-isomers are the only [m,n]-structures in which the tautomers with the labile H-atom residing at the pyridine N-atoms are the most stable. In the case of [2,4]-isomers, we have only two condensation forms, N9 and N8 (or N6 and N7), and for N9, the N9-H tautomer is the most stable; then, N2-H (4.0 kcal/mol) and finally, N4-H (5.6 kcal/mol), whereas for N8, the N8-H tautomer is the most stable; then, N4-H (4.8 kcal/mol) and finally, N2-H (6.3 kcal/mol).

### 2.2. Integral INICS Aromaticity

The aromaticity of the compounds composed of the pyridine and [m,n]diazepine rings can shed additional light on the stability and reactivity of these intricate groups of heteroaromatic compounds. Aromaticity has been evaluated in many ways [26], out of which the most popular are geometrical HOMA [27] and similar indices like HOMED [5,32,33,34] and NICS-derived indices [28,35,37,38,39,40,41]. Recently, Stanger introduced the integrated value of the NICSπZZ (∫ NICSπZZ) index [41], and Berger et al. showed such an integral NICS parameter to be related to the ring current via Ampère-Maxwell’s law [42]. We have established that out of NICS-like indices, the integral INICS index behaves the most reliably [44]. Therefore, here, we focus on variations of the integral INICS index and skip the discussion of all the other versions of NICS, which can be found in Tables S3–S8 in the Supplementary Materials file. Still, the values of INICS, which are negative, positive, and around zero, indicate aromaticity, antiaromaticity, and a lack of aromaticity, respectively. The NICSzz-scan curves of all studied pirydo[m,n]diazepines are presented in Figures S1–S6 in the Supplementary Materials file.

Let us start the NICS-scan analysis from the tautomers of the most stable N9 condensation isomer of the pirydo[1,3]diazepine system (Figure 1). Observe that, formally, in the six-membered pyrido and seven-membered [1,3]diazepine moieties of the N1-H or N3-H tautomer, there is, respectively, 6 and 8 π-electrons (including the free electron pair at the diazepine N1(-H) or N3(-H) atom) (Figure 1). On the other hand, formally, 7 π-electrons are present in each of the pyrido and [1,3]diazepine moieties of the N9-H tautomer (Figure 1). Thus, the pyrido and [1,3]diazepine rings in the N1-H and N3-H tautomers are expected to be aromatic and antiaromatic, respectively, whereas, in the N9-H tautomer, the two rings are probably antiaromatic. The conjecture stems from the fact that in the case of an odd number of electrons and (mostly) non-planar rings of an odd number of atoms, the formulas connecting aromaticity and antiaromaticity with the number of π-electrons cannot stem straightforward from the Hückel rules [77,78,79]. Moreover, when 7 + 7 π-electrons are shared by the fused six- and seven-membered heteroatomic rings of a nine-membered system, the heteroatoms’ positions and location of the mobile H atom significantly redistribute the π-electrons. Hence, the ring aromaticity/antiaromaticity cannot be predicted from the simple 4n + 2/4n rules.

According to the NICS_ZZ_ scan, it is immediately seen that the pyridine rings in the N1-H and N3-H tautomers are aromatic: the curves are directed to negative values (Figure 1a and Figure S2). In contrast, after location of the labile H-atom in the N9 position, the ring becomes strongly antiaromatic with the NICS scan directed toward positive values (Figure 1a). On the other hand, irrespectively the tautomer type, the NICS-curves of the [1,3]diazepine ring are directed towards positive values which mean that these rings are antiaromatic (Figure 1b). A closer look into the INICS values shows that for the pyridine ring and N1-H and N3-H tautomers, they are very close, −74.8 and −73.0 ppm·Å, while for the N9-H tautomer, it equals 278.3 ppm·Å. In contrast, for the [1,3]diazepine ring, the INICS values are equal to 102.5, 128.1, and 114.4 ppm·Å for the N1-H, N3-H, and N9-H tautomer, respectively.

Notice that the most stable N1-H tautomer has the lowest both values of INICS (−74.8; 102.5), the second has the second smallest first value and the highest second one (−73.0; 128.1), and the least stable one has a huge first value and intermediate second one (278.3; 102.5). However, the sum of the INICS values of the two rings agree qualitatively with the stability order: 27.7, 52.2, and 392.7 ppm·Å (Table 3) vs. 0.00, 5.63, and 11.95 kcal/mol (S 1).

In the most stable form of the pyrido[1,2]diazepines, the N1-H tautomer in the N9 condensation form, the pyridine ring is aromatic, as the INICS_ZZ_ = −75.6 ppm·Å and the NICS_ZZ_ plot has only negative values. On the other hand, the INICS_ZZ_ values for the N2-H and N6-H tautomers are 173.3 and 146.7 ppm·Å, respectively, and the curves go only through the positive values, indicating the ring antiaromaticity (Figure S1). Notice that atypically, the diazepine ring in the more stable N2-H tautomer is flat. The diazepine rings in all pyrido[1,2]diazepines are antiaromatic regardless of the type of tautomer and the type of condensation. For the [1,2]diazepine ring, the most considerable positive value of 580.5 ppm·Å is for the planar N2-H tautomer, and the intermediate for the N9-H tautomer is 287.5 ppm·Å, while the lowest value is 92.1 ppm·Å for the N1-H one. Considering the N9 condensation type, the sum of the INICS_ZZ_ values of the two rings agree qualitatively with the stability order: 16.5, 434.1, and 753.8 ppm·Å (Table 3) vs. 0.00, 27.70, and 19.34 kcal/mol (Table 1).

Irrespectively of the condensation type, the NICSzz-scans for the pyridine ring of the pyrido[1,4]diazepines reveal the aromatic nature only for the N1-H tautomers (INICS_ZZ_ = −55 ÷ 78 ppm·Å, Table 3). Thus, the pyridine ring in the remaining two N4-H and N6-H tautomers is antiaromatic (INICS_ZZ_ = 71.4 and 170.3 ppm·Å, respectively; Figure S3). For all tautomers and condensation types, the NICS_ZZ_ scan curves for the diazepine rings are positive, and they are antiaromatic. For the N9 condensation type, the INICSZZ([1,4]diazepine ring) = 136.6, 221.7, and 320.9 ppm·Å for the N1-H, N4-H, and N9-H tautomer, respectively. Unlike the [1,3] and [1,2] pyrido-diazepines, the agreement between the stability order and the sum of the INICS values occurs only for the N7 condensation type (Table 1 and Table 3). However, the lowest sum of the INICS always corresponds to the most stable tautomer of the given condensation type.

There is the equivalence of the N1-H and N5-H tautomers in pyrido[1,5]diazepines, for which the N6 and N9, as well as N7 and N8 condensation types, are also equivalent. The INICS values of the pyridine ring in the N1-H and N5-H are between −85 and −60 ppm·Å, indicating that they are aromatic. When the labile H atom is in the N6(N9) or N7(N8) position, the NICS_ZZ_ scans for the pyridine ring go through the positive values, and INICS_ZZ_ equals 133 and 144 ppm·Å, respectively (Table 3, Figure S4). The diazepine rings exhibit positive INICS_ZZ_ values for all tautomers, clearly indicating its antiaromaticity. The most stable N1-H(N5-H) tautomer for N9(N6) condensation has INICS_ZZ_ values equal to −60.3 ppm and 153.2 ppm·Å for pyridine and diazepine rings, respectively. The energetic stability order of the pyrido[1,5]diazepine tautomers is not reflected in the sums of the INICS_ZZ_ values, but the least stable isomer always has the highest INICS_ZZ_ value.

The NICS_zz_-scans for pirydo [2,3]diazepines show that in the case of N6 and N7 condensations, the pyridine ring is aromatic only for the N3-H tautomers (Figure S5). For the remaining tautomers and condensation types, the NICSzz scans of the pyridine and diazepine rings go only through the positive NICS_ZZ_ values. Although the INICS_ZZ_ values do not follow the stability order, it is worth noting that for the most stable N3-H tautomer and N6 condensation type and the second stable N3-H tautomer and N7 condensation type, the sums of the INICS_ZZ_ values of two rings are the lowest: 142 and 177 ppm·Å, respectively (Table 3).

In the case of pirydo [2,4]diazepine isomers, all NICS_zz_ scans go through the positive values, and all INICS_ZZ_ sum values are higher than 400 ppm·Å, showing that the rings are not aromatic (Figure S6). Interestingly, for each condensation type, the sum of the INICS_ZZ_ values agree qualitatively with the stability order (Table 2 and Table 3).

**Figure 2:**
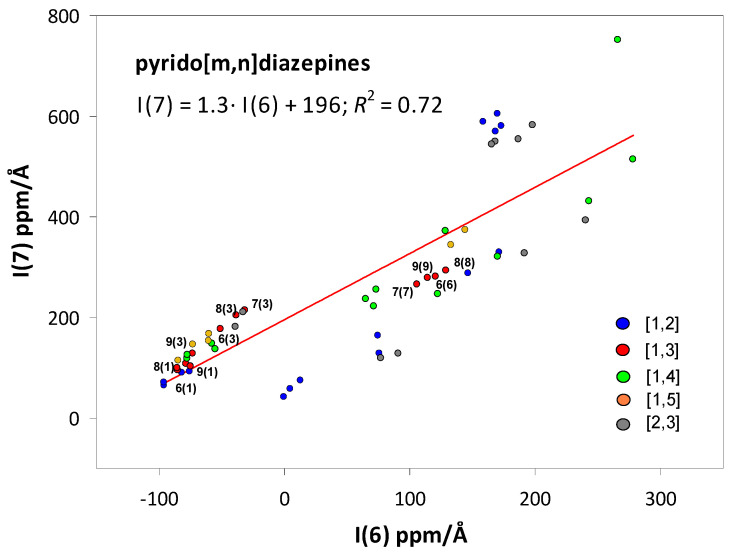
Linear trend between INICS_ZZ_ values of the 6− and 7−membered rings in pyrido[m,n]diazepines I(6) and I(7) (ppm·Å), respectively. [m,n] = [1,2], 1,3], [1,4], [1,5], and [2,3]. The values for pyrido[2,4]diazepines form a separate trend and were omitted. The k(h) notation stands for the Nk condensation type and Nh-H tautomer of the most stable pyrido[1,3]diazepine.

In the discussion, we have frequently mentioned that the energy stability of a given type of isomer/tautomer is qualitatively consistent with the INICS_ZZ_ values of the pyridine rings or, more often, the INICS_ZZ_ sum of both the six-member and seven-member rings. For this reason, we tested whether there is a general relationship between INICS_ZZ_ values and pyrido[m,n]diazepine stability. However, a comparison of the relative values of the molecular energies related to the energy of the most stable isomer, i.e., the N1-H N9 tautomer of condensed pyrido[1,3]diazepine (see total energies, Table S1), showed that no such general trend exists. Indeed, the square of the regression coefficient of the linear correlations between the relative energies and the INICS_ZZ_ values of pyridine, diazepine, and the sum of the two values is 0.45, 0.23, and 0.33, respectively. Nevertheless, there exists a weak linear correlation (*R*^2^ = 0.72) between the INICS_ZZ_ values of the pyridine I(6) and diazepine I(7) rings (Figure 2). This correlation is a consequence of the communication between the π-electron systems of the two rings. However, it is often hindered by the presence of single bonds around the Nk-H moiety in the diverse tautomers of pyrido[n,m]diazepines, which disrupts the continuity of the π-electron system and, therefore, weakens the correlation.

Let us now consider an important issue raised by the anonymous reviewers of this article. There are three possible ring currents in a fused two-ring system. Thus, in addition to the two indices characterizing the six- and seven-member rings, (I(R6) and I(R7), respectively (Table 3), a third one that describes the ring current through the entire system should be provided. In the manuscript, we use I_SUM_ = I(R6) + I(R7) for a supporting description of the aromaticity of the entire system. Still, we know that it cannot be taken as a rigorous measure of the ring current through the rim of a condensed two-ring system because this index silently assumes the additive contribution of the two rings. Yet, the problem of estimating the index for the rim over two rings is complex. If it could be easy, the solution would already be given in the Berger et al. paper [42], demonstrating physical justification for the NICS integral parameter based on Ampère–Maxwell’s law. However, in the article, only planar and symmetrical (convex) rings have been considered: cyclobutadiene, benzene, cyclohexadiene, borazine, para-benzoquinone, hexadehydro annulene, and central rings of porphyrin and isophlorin.

Nevertheless, the situation is already complicated for convex non-planar rings, for which INICS integrals from the two sides of the ring are different [44,80]. If we ignore the joining bond between seven and six-membered rings in pyridodiazepines, we have a non-convex and non-planar 11-membered ring (cyclic system). In classical electrodynamics, mainly topology matters [81], while in molecular (quantum) systems, the INICS integral depends on the position of the normal along which the integral is calculated (Figure S10). For the normal straight lines through the center of the benzene plane, the point in the middle from the center to the bond center, and the middle from the center to the C-atom, the INICSs are equal to −145.63, −170.96, and −165.77 (ppm·Å), respectively. Now consider a non-convex and non-flat 11-member ring. Finding the center and a straight line passing through is easy. It would likely be near the connecting CC bond. Still, we cannot convincingly conclude that scanning this normally is the right way to reflect the ring current in an 11-member cycle. Finding the single NICS-derived index condition for two-ring and multi-ring systems is intricate for a large separate project. There is hope though, as the authors of the paper [42] announced the development of an integration scheme based on Ampère–Maxwell’s law, including an analysis of the stagnation graphs, which will make it possible to determine all the current strengths in the molecule.

### 2.3. INICS vs. Electron Density Parameters at the Ring Critical Points

**Figure 3:**
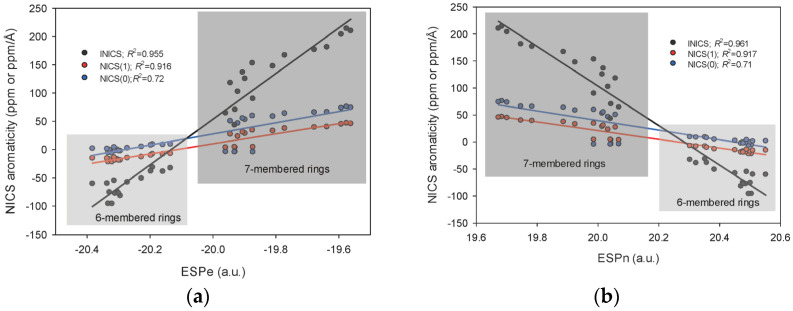
Linear correlations between Electrostatic Potential in Ring Critical Points NICS aromaticity indices INICS, NICS(1), and NICS(0) of the 6− and 7−membered rings in pyrido[m,n]diazepines and (**a**) ESPe (Electrostatic Potential from Electrons), and (**b**) ESPn (Electrostatic Potential from Nuclei).

Thanks to a suggestion of an anonymous reviewer, we performed the Atoms-In-Molecules (AIM) analysis [82,83] and determined the values of various electron density parameters at the Ring Critical Points (RCPs). We followed the work of M. Palusiak and T. M. Krygowski [84]. The comparison of INICS with AIM parameters in RCP appeared unexpected, significant, and worth adding to the manuscript. The following parameters in RCP were considered: the Rho (Electron Density), Laplacian of Rho (Trace of Hessian of Rho), V (Virial Field = Potential Energy Density), G (Lagrangian Form of Kinetic Energy Density, K (Hamiltonian Form of Kinetic Energy Density), H (H = G + V, Total Energy), L = K − G = Lagrangian Density, ESP (Total Electrostatic Potential), ESPe (Electrostatic Potential from Electrons), and ESPn (Electrostatic Potential from Nuclei) using AIMAll Software [85]. We expected that the best correlations would be obtained with energy parameters, as the Palusiak and Krygowski work concluded, even though in their study, the correlations were much stronger for the HOMA than for the NICS-type parameters. Surprisingly, for the INICS descriptor applied to the six- and seven-membered rings of pyridodiazepines, the only well-correlating parameters appeared to be the Electrostatic Potentials from the electrons and nuclei ESPe and ESPn [86], which Palusiak and Krygowski did not previously consider. As before, the correlations with INICS are stronger than those with NICS(1), which are stronger than those with NICS(0) (Figure 3).

**Figure 4:**
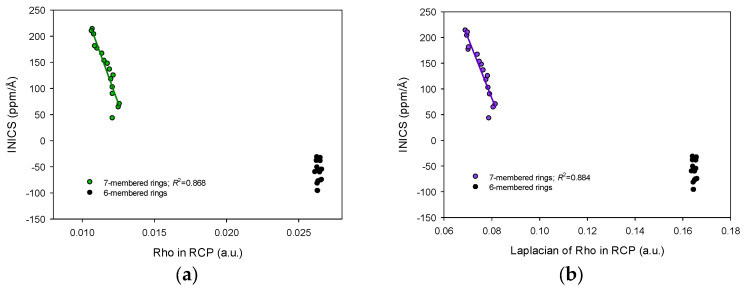
Linear correlations between the electron density and Laplacian of electron density in Ring Critical Points and INICS aromaticity index of the 7−membered rings in pyrido[m,n]diazepines and lack of such a correlation for the 6−membered rings: (**a**) electron density Rho, and (**b**) Laplacian of electron density ∇^2^(Rho). Two points of [2,3]− and two of [2,4]−isomers deviate from trends and are omitted.

**Figure 5:**
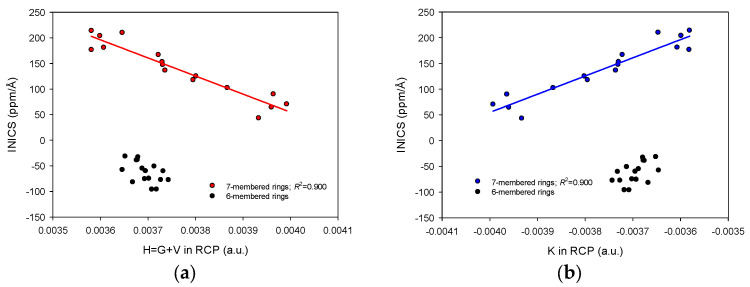
Linear correlations between energies in Ring Critical Points and INICS aromaticity index of the 7−membered rings in pyrido[m,n]diazepines and lack of such a correlation for the 6−membered rings: (**a**) Total Energy H (H = G + V), and (**b**) Hamiltonian Form of Kinetic Energy Density K. Two points of [2,3]− and two of [2,4]−isomers deviate from trends and are omitted.

On the other hand, the relationships between INICS and any other parameters in RCP were split into two parts (Figure 4 and Figure 5). The one for the six-membered ring was more or less constant, and the other for the seven-membered rings varied and linearly correlated with INICS. The above means that the Rho and Laplacian of Rho in RCP (Figure 4), and the H and K in RCP (Figure 5) (as well as V, G, K, and L in RCP) of the six-membered ring of pyridodiazepines practically do not change with the diazepine type and the labile proton position (Figure 4 and Figure 5). In contrast, for the seven-membered rings, the two factors are sufficient to modify the AIM parameters in RCP and display aromaticity changes in the antiaromatic rings (Figure 4). It should be noted that the electrostatic potentials of electrons and nuclei ESPe and ESPn can uniformly reveal aromaticity changes in both aromatic and antiaromatic systems (Figure 3).

## 3. Methods

The structures were optimized using the DFT B3LYP functional [87,88] and Dunning’s correlation consistent cc-pVTZ basis set [89,90,91]. All optimized structures exhibited all real harmonic frequencies. All calculations were performed using the Gaussian 09, Rev. D.01, suite of programs [92]. The isomers’ energetics and population were calculated according to the total and Gibbs’ free energies (Supporting Information, Tables S1 and S2). Hereafter, populations estimated for 298.15 K were calculated based on Gibbs free energy differences between conformers and the Maxwell–Boltzmann distribution equation. The gauge-invariant calculation of the nuclear magnetic shielding constants [93] has been used. The in-house ARONICS [45] computer program was used for the NICS scan and NICS indices calculations. For an optimized molecule with non-planar rings, ARONICS creates a Gaussian input file for calculating NMR shielding constants and plotting the NICS scan. The program finds the rings in the input structure itself and determines the optimal plane for non-planar rings by the least squares fitting applied to the coordinates of the ring’s heavy atoms and the vector normal to this plane at the center of the ring. The probe points are placed along the normal straight line, and the step size can vary for different intervals. In this study, the step was 0.1 Å for ⟨−5; 5⟩ and 0.3 Å for ⟨−9.8, −5.0⟩∪⟨5.0, 9.8⟩, where the interval limits denote distances from the ring center. NMR calculations can then be performed for each ring, after which ARONICS reads the output files and returns the NICS values at all probe points, the NICS scan plot, and the NICS scan integral, INICS. Note that for INICS, we use the unit ppm·Å, which can be replaced by an SI-based unit, as consistently proposed in Ref. [42]. However, since ppm is widely used in NMR and NICS studies, and ångström is a very convenient unit at the molecular level, we want to stay closer to chemical practice than purist formalism. We also recalculated all systems using the solvent’s polarized continuum model (PCM), taking water as the surrounding medium (Tables S12–S17 in Supplementary Information file). We correlated the molecules’ energies (Figure S2) and NICS descriptors (Figure S3) in the gas phase and aqueous solution. The linear correlations between the NICS(0), NICS(I), and INICS descriptors of the molecules in the gas phase and water are very good: *R*^2^ ≥ 0.997 for energies and ≥0.92 for aromaticity indices. However, the correlations are the best for INICS (*R*^2^ = 0.989) and the least good for NICS(0) (*R*^2^ = 0.920). The high quality of the correlations makes a separate discussion of the solvent effect unnecessary. Ultimately, we performed the reference DFT calculations as the most popular, and the routinely used semi-empirical global hybrid B3LYP functional has several known drawbacks [94,95]. We calculated all systems with a long-range-corrected CAM-B3LYP functional [96,97] and BHandHlyp (Becke-Half-and-Half-LYP) functional with a 1:1 mixture of DFT and exact exchange, which performs well in several medium sized organic systems [98,99]. Eventually, we achieved satisfactory agreement between the results found using all three functionals (Figure S1). In particular, the energy differences between tautomers predicted using the B3LYP functional agreed with those of the two others. Therefore, we can state that conclusions derived based on the B3LYP functional are semi-quantitatively valid for a larger group of hybrid functionals.

## 4. Conclusions

The structure and energetics of the constitutional isomers and tautomers of pyrido[m,n]diazepines (m = 1, 2; n = 2, 3, 4, 5; m ≠ n) were studied at the B3LYP/cc-pVTZ level. In the pyridine fused with the diazepine ring, the position of the N occupied all but the junction positions between the rings. The two N-atoms in the diazepine seven-membered rings occupied two out of five locations. For all constitutions of the heavy atoms, three locations of the labile H atom were considered: at one pyridine and two diazepine N atoms. Some configurations of the nitrogen atoms in the pyrido[m,n]diazapine system appeared to be equivalent because of the molecular symmetry. In total, more than 100 isomers were considered because, for some molecules, there were some other conformers, including entirely flat ones.

The stability of pyrido[m,n]diazepines was analyzed in the following sequence: for the given location of the N-atoms in the [m,n]diazepine ring, the condensation type, i.e., position of the N-atom in the pyridine ring was taken into account, and then three tautomers were considered. The stability order was established against the most stable tautomers of the most stable condensation types of given [m,n]diazepines. Pyrido[1,3]diazepines appeared the most, and pyrido[2,4]diazepines the least stable (ca. 26 kcal/mol). In the pyrido[1,n]diazepine group (n = 2–5), the [1,5], [1,4], and [1,2] isomers are higher in energy by ca. 4.5, 7.0, and 20.0 kcal/mol, respectively. All the most stable pyrido[1,n]diazepines are condensed similarly: the N-atoms are near the ring’s junction bond but on opposite sites. Again, the most stable [2,n]-forms are those with the pyridine ring N6-atom near the junction bond, but in the case of [2,3]-isomers, the labile H atom is positioned at the N3-atom, whereas in the case of [2,4]-isomers, it is located at the same pyridine N6 atom.

Surprisingly, for the [1,2]-, [1,3]-, and [1,4]-isomer condensation types, the same N9 > N7 > N6 > N8 stability pattern obeys, while for the [1,5]-type, it is simplified as N9(=N6) > N7(=N8) due to symmetry. The Gibbs free energy differences are as follows: (0.0, 4.2, 4.9, 6.2); (0.0, 3.6, 3.9, 4.8); (0.0, 5.0; 6.6, 7.5); and (0.0, 5.9) kcal/mol, for [1,2]-, [1,3]-, [1,4]-, and [1,5]-isomers, respectively. For the [2,3]-isomers, the stability sequence is N6 > N7 > N9 > N8, and energy differences: 0.0, 1.8, 17.9, and 18.2 kcal/mol, respectively. For the [2,4]-isomers, the N7(N8) condensations are more stable than the N6(N9) ones by 26.2 kcal/mol. The stability order of the tautomers for a given diazepine ring is more complex.

The ring’s aromaticity in the pyridine[m,n]diazepines was established for pirydo [m,n]diazepines based on the integral INICS index [44], which results from the NICSzz-scan curves’ integration. The relation to the ring current physically justifies the integral INICS index via Ampère–Maxwell’s law [42]. According to the NICS_ZZ_ scans, the six-membered pyrido rings have negative INICS_ZZ_ values and can be aromatic only if not protonated at the N-atom. All protonated pyrido rings exhibit meaningful positive INICS_ZZ_ values and can be assigned as antiaromatic. However, some non-protonated pyrido rings also have substantial positive INICS_ZZ_ indices and are antiaromatic. This is the case of the tautomers of pyrido[1,2]diazepine and pyrido[1,4]diazepine other than the N1-H ones, all pyrido[2,4]diazepine isomers, and all but two pyrido[2,3]diazepine isomers. All seven-membered rings in the studied molecules have large positive INICS_ZZ_ values and thus can be described as antiaromatic. The comparison of the INICS values for the six- and seven-membered rings reveals a weak linear correlation (*R*^2^ = 0.72), displaying communication between the π-electron systems of the two rings.

The Atoms-In-Molecules (AIM) analysis provided the values of various electron density parameters at the Ring Critical Points (RCPs) demonstrated by M. Palusiak and T. M. Krygowski to be excellent aromaticity measures [84]. The comparison of INICS with AIM parameters in RCP like Electron Density, its Laplacian, energies of different types V, G, K, H (H = G + V, L = K – G), and the Electrostatic Potentials from Electrons and from Nuclei (ESPe and ESPn), demonstrated that only the latter two (not previously considered [84]) provided good correlations. Thus, the electrostatic potentials of electrons and nuclei, ESPe and ESPn, can uniformly reveal aromaticity changes in both aromatic and antiaromatic systems. On the other hand, the relationships between INICS and any other parameters in RCP were split into two parts: one for the six- and the other for the seven-membered ring. The former was more or less constant, and the other varied and linearly correlated with INICS. The above means that the six-membered ring of pyridodiazepines practically do not change with the diazepine type and the labile proton position, whereas the seven-membered rings, are sensitive to such structural modifications, and the AIM parameters in RCP and display aromaticity changes in the antiaromatic rings.

## Figures and Tables

**Table 1:** The energy differences and relative Gibbs free energies ΔG_PDZ_, ΔG_T_, ΔE_PC_, ΔG_PC_ (kcal/mol) are referred to, respectively, by the most stable isomer of pyrido[1,n]diazepines n = 2,3,4,5 pyridine condensation (PC) in a given diazepine type (DT), tautomer type (T) in particular types of pyridine condensation, and planarity (P) bicyclic system of np (non-planar) and p and (planar) tautomers.

	PC	T	P	ΔG_PDZ_	ΔG_PC_	ΔE_T_	ΔG_T_		PC	T	P	ΔG_PDZ_	ΔG_PC_	ΔE_T_	ΔG_T_
**pyrido[1,2]diazepines**	N6	N1-H	n		4.89	0.00	0.00	**pirydo[1,4]diazepinese**	N6	N1-H	n		6.63	0.00	0.00
		N2-H	n			19.64	19.03			N4-H	n			19.98	19.40
		N2-H	p			22.09	21.38			N6-H	n			18.63	17.16
		N6-H	n			36.20	34.53		N7	N1-H	n		5.00	0.00	0.00
	N7	N1-H	n		4.22	0.00	0.00			N4-H	p			20.39	19.51
		N2-H	n			18.89	18.63			N4-H	n			20.20	19.51
		N7-H	n			22.44	21.33			N7-H	n			15.05	14.37
	N8	N1-H	n		6.17	0.00	0.00		N8	N1-H	n		7.45	0.00	0.00
		N2-H	p			19.69	19.15			N4-H	n			22.86	22.04
		N2-H	n			23.56	22.69			N8-H	p			19.06	17.37
		N8-H	n			27.69	26.39								
	N9	N1-H	n	19.74	0.00	0.00	0.00		N9	N1-H	n	7.13	0.00	0.00	0.00
		N2-H	p			25.61	24.75			N4-H	n			27.60	26.63
		N2-H	n			28.71	27.78			N9-H	p			13.03	12.02
		N9-H	n			21.08	19.34								
**pyrido [1,3]diazepines**	N6	N1-H	n			2.06	2.37	**pirydo [1,5]diazepines**	N6	N1-H	n			8.06	7.73
		N3-H	n		3.86	0.00	0.00			N5-H	n	4.54	0.00	0.00	0.00
		N6-H	n			16.70	16.16			N6-H	p			11.65	10.82
	N7	N1-H	n			0.52	1.84		N7	N1-H	n		5.92	0.00	0.00
		N3-H	n		3.62	0.00	0.00			N5-H	n			0.58	0.66
		N7-H	n			16.49	16.90			N7-H	p			13.18	12.12
	N8	N1-H	n			1.07	1.75		N8	N1-H	p			0.58	0.66
		N3-H	n		4.83	0.00	0.00			N5-H	n		5.92	0.00	0.00
		N8-H	n			17.96	17.85			N8-H	p			13.18	12.12
	N9	N1-H	n	0.00	0.00	0.00	0.00		N9	N1-H	n		0.00	0.00	0.00
		N3-H	n			6.10	5.63			N5-H	n			8.06	7.73
		N9-H	n			12.64	11.95			N9-H	n			11.65	10.82

**Table 2:** The energy differences and relative Gibbs free energies ΔG_PDZ_, ΔG_T_, ΔE_PC_, ΔG_PC_ (kcal/mol) are referred to, respectively, by the most stable isomer of pyrido[2,n]diazepines n = 3, 4, pyridine condensation (PC) in a given diazepine type (DT), tautomer type (T) in particular types of pyridine condensation, and planarity (P) bicyclic system of np (non-planar) and p and (planar) tautomers.

	PC	T	P	ΔG_PDZ_	ΔG_PC_	ΔE_T_	ΔG_T_		PC	T	P	ΔG_PDZ_	ΔG_PC_	ΔE_T_	ΔG_T_
**pyrido[2,3]diazepines**	N6	N2-H	p			18.64	18.63	**pirydo[2,4]diazepinese**	N6	N2-H	p			5.58	5.42
		N3-H	n	22.81	0.00	0.00	0.00			N4-H	n			3.97	4.24
		N6-H	n			21.90	21.15			N6-H	p	26.16	0.00	0.00	0.00
	N7	N2-H	p			18.22	18.47		N7	N2-H	p			4.77	4.75
		N3-H	n		1.77	0.00	0.00			N4-H	p			6.26	6.32
		N7-H	n			30.11	28.89			N7-H	n		0.05	0.00	0.00
	N8	N2-H	n		18.23	0.00	0.00		N8	N2-H	p			6.26	6.32
		N3-H	p			1.93	2.01			N4-H	p			4.77	4.75
		N8-H	n			4.57	3.76			N8-H	n		0.05	0.00	0.00
	N9	N2-H	n		17.89	0.00	0.00		N9	N2-H	n			3.97	4.24
		N3-H	p			0.83	0.74			N4-H	p			5.58	5.42
		N9-H	p			11.38	9.71			N9-H	p	26.16	0.00	0.00	0.00

**Table 3:** The INICS values of pyrido[m,n]diazepines m = 1, 2; n = 2, 3, 4, 5; m ≠ n, pyridine for pyridine (R6) and diazepine (R7) rings. Only the most stable form of a given tautomer is taken into account.

	PC	T	I(R6)	I(R7)	I_SUM_		PC	T	I(R6)	I(R7)	I_SUM_		PC	T	I(R6)	I(R7)	I_SUM_
**pyrido[1,2]diazepines**	N6	N1-H	−95.9	64.5	−31.4	**pyrido[1,3]diazepines**	N6	N1-H	−85.0	94. 6	9.5	**pyrido[1,4]diazepines**	N6	N1-H	−77.5	118.0	40.4
		N2-H	158.8	588.8	747.6			N3-H	−50.9	176.8	125.9			N4-H	73.4	255.0	328.4
		N6-H	171.5	329.4	501.0			N6-H	120.8	280.9	401.8			N6-H	243.2	431.0	674.2
	N7	N1-H	−81.7	90.1	8.3		N7	N1-H	−78.4	107.6	29.1		N7	N1-H	−57.7	147.8	90.2
		N2-H	168.6	569.4	738.0			N3-H	−31.4	214.0	182.5			N4-H	128.8	371.9	500.8
		N7-H	74.7	163.5	238.2			N7-H	106.0	265.1	371.1			N7-H	122.6	246.4	369.0
	N8	N1-H	−96.0	70.5	−25.5		N8	N1-H	−85.3	99.0	13.7		N8	N1-H	−77.3	125.3	48.0
		N2-H	170.2	604.5	774.6			N3-H	−38.3	204.0	165.7			N4-H	65.0	236.1	301.1
		N8-H	75.7	128.2	203.8			N8-H	129.1	293.0	422.2			N8-H	278.3	514.2	792.5
	N9	N1-H	−75.6	92.1	16.5		N9	N1-H	−74.8	102.5	27.7		N9	N1-H	−55.0	136.6	81.6
		N2-H	173.3	580.5	753.8			N3-H	−73.0	128.1	55.2			N4-H	71.4	221.7	293.1
		N9-H	146.7	287.5	434.1			N9-H	278.3	114.4	392.7			N9-H	170.3	320.9	491.2
**pyrido[1,5]diazepines**	N6	N1-H	−84.5	114.1	29.7	**pyrido[2,3]diazepines**	N6	N2-H	186.9	554.0	740.9	**pyrido[2,4]diazepines**		N2-H	808.0	325.1	1133.0
		N5-H	−60.3	153.2	92.9			N3-H	−38.9	181.2	142.3		N6	N4-H	651.9	246.4	898.3
		N6-H	133.1	343.9	477.0			N6-H	91.0	127.9	218.9			N6-H	278.7	230.5	509.2
	N7	N1-H	−60.0	167.0	107.0		N7	N2-H	168.4	549.5	717.9			N2-H	820.6	331.3	1151.8
		N5-H	−72.9	146.1	73.2			N3-H	−32.8	210.2	177.4		N7	N4-H	913.7	383.9	1297.6
		N7-H	144.4	373.5	518.0			N7-H	191.8	327.3	519.2			N7-H	252.1	187.4	439.4
	N8	N1-H	−72.9	146.1	73.2		N8	N2-H	198.2	582.1	780.3			N2-H	913.7	383.9	1297.6
		N5-H	−60.0	167.0	107.0			N3-H	168.4	549.5	717.9		N8	N4-H	820.6	331.3	1151.8
		N8-H	144.4	373.5	518.0			N8-H	77.0	118.8	195.8			N8-H	252.1	187.4	439.4
	N9	N1-H	−60.3	153.2	92.9		N9	N2-H	165.6	543.7	709.4			N2-H	651.9	246.4	898.3
		N5-H	−84.5	114.1	29.7			N3-H	186.9	186.9	373.9		N9	N4-H	807.9	325.2	1133.1
		N9-H	133.1	343.9	477.0			N9-H	240.5	392.9	633.4			N9-H	278.7	230.5	509.2

## Data Availability

All data generated in this study are presented in the Manuscript and its Supplementary Materials.

## Supplementary Materials

The following supporting information can be downloaded at: https://www.mdpi.com/article/10.3390/molecules28155684/s1. Figure S1: The correlations between energy differences ΔE (kcal/mol) of pyrido[m,n]diazepines calculated using the B3LYP, CAM-B3LYP, and BHandHLYP functional; Figure S2: The correlations between relative (a) energy ΔE and (b) relative Gibbs free energy ΔG (kcal/mol) of pyrido[m,n]diazepines estimated using the B3LYP/cc-pVTZ method in the gas phase and water simulated using a PCM model; Figure S3: The correlations between NICS parameters of rings in pyrido[m,n]diazepines estimated using the B3LYP/cc-pVTZ method in the gas phase and water simulated using a PCM model; Figure S4: The NICSzz-scans vs. distance from the ring center along the normal to the ring for the pirydo[1,2]diazepines; Figure S5: The NICSzz-scans vs. distance from the ring center along the normal to the ring for the pirydo[1,3]diazepines; Figure S6: The NICSzz-scans vs. distance from the ring center along the normal to the ring for the pirydo[1,4]diazepines; Figure S7: The NICSzz-scans vs. distance from the ring center along the normal to the ring for the pirydo[1,5]diazepines; Figure S8: The NICSzz-scans vs. distance from the ring center along the normal to the ring for the pirydo[2,3]diazepines; Figure S9: The NICSzz-scans vs. distance from the ring center along the normal to the ring for the pirydo[2,4]diazepines; Figure S10: The NICS curves taken for the normal straight lines through different points of the benzene plane; Table S1: Energy and relative Gibbs free energies of pyrido[1,n]diazepines n = 2,3,4,5; Table S2: Energy and relative Gibbs free energies of pyrido[2,n]diazepines n = 3,4; Table S3: The energies and energy differences ΔE (kcal/mol) referred to the most stable diazepine type isomers of pyrido[m,n]diazepines calculated using different functionals: B3LYP, CAM-B3LYP, BHandHLYP; Table S4: The energies and energy differences ΔE (kcal/mol) referred to the globally most stable isomer of pyrido[m,n]diazepines calculated using different functionals: B3LYP, CAM-B3LYP, BHandHLYP; Table S5: The energies and Gibbs free energy differences ΔG (kcal/mol) referred to the most stable diazepine type isomers of pyrido[m,n]diazepines calculated using different functionals: B3LYP, CAM-B3LYP, BHandHLYP; Table S6: Different NICS aromaticity indices of ring in pyrido[1,2]diazepines in the gas phase; Table S7: Different NICS aromaticity indices of ring in pyrido[1,3]diazepines in the gas phase; Table S8: Different NICS aromaticity indices of ring in pyrido[1,4]diazepines in the gas phase; Table S9: Different NICS aromaticity indices of ring in pyrido[1,5]diazepines in the gas phase; Table S10: Different NICS aromaticity indices of ring in pyrido[2,3]diazepines in the gas phase; Table S11: Different NICS aromaticity indices of ring in pyrido[2,4]diazepines in the gas phase; Table S12: Different NICS aromaticity indices of ring in pyrido[1,2]diazepines in the water phase; Table S13: Different NICS aromaticity indices of ring in pyrido[1,3]diazepines in the water phase; Table S14: Different NICS aromaticity indices of ring in pyrido[1,4]diazepines in the water phase; Table S15: Different NICS aromaticity indices of ring in pyrido[1,5]diazepines in the water phase; Table S16: Different NICS aromaticity indices of ring in pyrido[2,3]diazepines in the water phase; Table S17: Different NICS aromaticity indices of ring in pyrido[2,4]diazepines in the water phase.

## Sample Availability

All calculation files are available from the authors.
