# Supplementary material for: On Integral INICS Aromaticity of Pyridodiazepine Constitutional Isomers and Tautomers
Source: Molecules. 2023 Jul 27;28(15):5684. doi: 10.3390/molecules28155684 (PMC10419959; doi:10.3390/molecules28155684)
Supplement: Supplementary file 1 [file molecules-28-05684-s001.zip › molecules-2443585-supplementary.pdf]

Supplementary Materials to

On integral NICS aromaticity of pyridodiazepine constitutional isomers and tautomers

Małgorzata Jarończyk[1,]*, Sławomir Ostrowski[2], and Jan Cz. Dobrowolski[1,2,]*

[1]National Medicines Institute, 30/34 Chełmska Street, 00-725 Warsaw, Poland

[2]Institute of Chemistry and Nuclear Technology, 16 Dorodna Street, 03-195 Warsaw, Poland; s.ostrowski@ichtj.waw.pl

*Correspondence: m.jaronczyk@nil.gov.pl (M.J.); j.dobrowolski@nil.gov.pl (J.C.D.)

	CONTENT	PAGE
Table S1	Energy and relative Gibbs free energies of pyrido[1,n]diazepines n=2,3,4,5	2
Table S2	Energy and relative Gibbs free energies of pyrido[2,n]diazepines n=3,4	3
Table S3	The energies and energy differences ΔE (kcal/mol) referred to **the most stable diazepine type isomers of pyrido[m,n]diazepines** calculated using different functionals: B3LYP, CAM-B3LYP, BHandHLYP	4
Table S4	The energies and energy differences ΔE (kcal/mol) referred to **the globally most stable isomer of pyrido[m,n]diazepines** calculated using different functionals: B3LYP, CAM-B3LYP, BHandHLYP	5
Table S5	The energies and Gibbs free energy differences ΔG (kcal/mol) referred to **the most stable diazepine type isomers of pyrido[m,n]diazepines** calculated using different functionals: B3LYP, CAM-B3LYP, BHandHLYP	6
Figure S1	The correlations between energy differences ΔE (kcal/mol) of pyrido[m,n]diazepines calculated using the B3LYP, CAM-B3LYP, and BHandHLYP functionals	7
Figure S2	The correlations between relative (a) energy ΔE and (b) relative Gibbs free energy ΔG (kcal/mol) of pyrido[m,n]diazepines estimated using the B3LYP/cc-pVTZ method in the gas phase and water simulated using a PCM model.	8
Figure S3	The correlations between NICS parameters of rings in pyrido[m,n]diazepines estimated using the B3LYP/cc-pVTZ method in the gas phase and water simulated using a PCM model	8
Figure S4	The NICSzz-scans vs. distance from the ring center along the normal to the ring for the pirydo[1,2]diazepines	9
Figure S5	The NICSzz-scans vs. distance from the ring center along the normal to the ring for the pirydo[1,3]diazepines	10
Figure S6	The NICSzz-scans vs. distance from the ring center along the normal to the ring for the pirydo[1,4]diazepines	11
Figure S7	The NICSzz-scans vs. distance from the ring center along the normal to the ring for the pirydo[1,5]diazepines	12
Figure S8	The NICSzz-scans vs. distance from the ring center along the normal to the ring for the pirydo[2,3]diazepines	13
Figure S9	The NICSzz-scans vs. distance from the ring center along the normal to the ring for the pirydo[2,4]diazepines	14
Figure S10	The NICS curves taken for the normal straight lines through different points of the benzene plane.	15
Table S6	Different NICS aromaticity indices of ring in pyrido[1,2]diazepines in the gas phase.	16
Table S7	Different NICS aromaticity indices of ring in pyrido[1,3]diazepines in the gas phase.	17
Table S8	Different NICS aromaticity indices of ring in pyrido[1,4]diazepines in the gas phase.	18
Table S9	Different NICS aromaticity indices of ring in pyrido[1,5]diazepines in the gas phase.	19
Table S10	Different NICS aromaticity indices of ring in pyrido[2,3]diazepines in the gas phase.	20
Table S11	Different NICS aromaticity indices of ring in pyrido[2,4]diazepines in the gas phase.	21
Table S12	Different NICS aromaticity indices of ring in pyrido[1,2]diazepines in the water phase.	22
Table S13	Different NICS aromaticity indices of ring in pyrido[1,3]diazepines in the water phase.	23
Table S14	Different NICS aromaticity indices of ring in pyrido[1,4]diazepines in the water phase.	24
Table S15	Different NICS aromaticity indices of ring in pyrido[1,5]diazepines in the water phase.	25
Table S16	Different NICS aromaticity indices of ring in pyrido[2,3]diazepines in the water phase.	26
Table S17	Different NICS aromaticity indices of ring in pyrido[2,4]diazepines in the water phase.	27

Table S1. The energy and relative Gibbs free energies E, G (hartree) are referred to respectively by the most stable isomer of pyrido[1,n]diazepines n=2,3,4,5 pyridine condensation (PC) in a given diazepine type (DT), tautomer type (T) in particular types of pyridine condensation, and planarity (P) bicyclic system of np (non-planar) and p and (planar) tautomers.

DT	PC	T	P	E_T	G_T	DT	PC	T	P	E_T	G_T
pyrido[1,2]diazepines	N6	N1-H	n	-473.419641	-473.312429	pirydo[1,4]diazepinese	N6	N1-H	n	-473.436405	-473.329775
		N2-H	n	-473.388340	-473.282096			N4-H	n	-473.404559	-473.298856
		N2-H	p	-473.384443	-473.278351			N6-H	n	-473.406722	-473.302422
		N6-H	n	-473.361958	-473.257394		N7	N1-H	n	-473.439271	-473.332369
	N7	N1-H	n	-473.420807	-473.313502			N4-H	p	-473.406786	-473.301270
		N2-H	n	-473.390696	-473.283807			N4-H	n	-473.407080	-473.301271
		N7-H	n	-473.385048	-473.279509			N7-H	n	-473.415294	-473.309469
	N8	N1-H	n	-473.417710	-473.310392		N8	N1-H	n	-473.435263	-473.328464
		N2-H	p	-473.386324	-473.279876			N4-H	n	-473.398830	-473.293337
		N2-H	n	-473.380159	-473.274226			N8-H	p	-473.404896	-473.300784
		N8-H	n	-473.373586	-473.268337						
	N9	N1-H	n	-473.427552	**-473.320232**		N9	N1-H	n	-473.447418	**-473.340341**
		N2-H	p	-473.386736	-473.280792			N4-H	n	-473.403441	-473.297897
		N2-H	n	-473.381807	-473.275960			N9-H	p	-473.426656	-473.321178
		N9-H	n	-473.393962	-473.289404						
pyrido[1,3]diazepines	N6	N1-H	n	-473.450275	-473.343002	pirydo[1,5]diazepines	N6	N1-H	n	-473.438714	-473.332136
		N3-H	n	-473.451104	-473.345928			N5-H	n	-473.451552	**-473.344459**
		N6-H	n	-473.424826	-473.319000			N6-H	p	-473.432992	-473.327217
	N7	N1-H	n	-473.448427	-473.341208		N7	N1-H	n	-473.441778	-473.335026
		N3-H	n	-473.450125	-473.343989			N5-H	n	-473.440851	-473.333982
		N7-H	n	-473.421496	-473.315538			N7-H	p	-473.420768	-473.315713
	N8	N1-H	n	-473.459200	**-473.351702**		N8	N1-H	p	-473.440851	-473.333982
		N3-H	n	-473.449486	-473.342735			N5-H	n	-473.4417778	-473.335026
		N8-H	n	-473.439059	-473.332658			N8-H	p	-473.4207675	-473.315713
	N9	N1-H	n	-473.450275	-473.343002		N9	N1-H	n	-473.4515516	**-473.344459**
		N3-H	n	-473.451104	-473.345928			N5-H	n	-473.4387136	-473.332136
		N9-H	n	-473.424826	-473.319000			N9-H	n	-473.432992	-473.327217

Table S2. The energy and relative Gibbs free energies E, G (hartree) are referred to respectively by the most stable isomer of pyrido[2,n]diazepines n=3,4, pyridine condensation (PC) in a given diazepine type (DT), tautomer type (T) in particular types of pyridine condensation, and planarity (P) bicyclic system of np (non-planar) and p and (planar) tautomers.

	PC	T	P	E_T	G_T		PC	T	P	E_T	G_T
**pyrido[2,3]diazepines**		N2-H	p	-473.392485	-473.285646	**pirydo[2,4]diazepinese**		N2-H	p	-473.400382	-473.295595
		N3-H	n	-473.422189	**-473.315340**			N4-H	n	-473.402939	-473.297486
	N6	N6-H	n	-473.387289	-473.281632		N6	N6-H	p	-473.409271	**-473.304237**
		N2-H	p	-473.390241	-473.283079			N2-H	p	-473.401947	-473.296579
		N3-H	n	-473.419272	-473.312509			N4-H	p	-473.399583	-473.294084
	N7	N7-H	n	-473.371283	-473.266463		N7	N7-H	n	-473.409555	-473.304151
		N2-H	n	-473.393320	-473.286276			N2-H	p	-473.399583	-473.294084
		N3-H	p	-473.390241	-473.283079			N4-H	p	-473.401947	-473.296579
	N8	N8-H	n	-473.386042	-473.280282		N8	N8-H	n	-473.409555	-473.304151
		N2-H	n	-473.393804	-473.286833			N2-H	n	-473.402939	-473.297486
		N3-H	p	-473.392485	-473.285646			N4-H	p	-473.400382	-473.295594
	N9	N9-H	p	-473.375663	-473.271364		N9	N9-H	p	-473.409271	**-473.304237**

Table S3. The energies and energy differences ΔE (kcal/mol) referred to **the most stable diazepine type isomers of pyrido[m,n]diazepines** (m=1, n=2-5; m=2, n=3,4) calculated using different functionals: B3LYP, CAM-B3LYP, BHandHLYP where PC denotes pyridine condensation type, DT is a given diazepine type, and T is tautomer type.

	PC	(T)	E_{B3LYP}	$E_{CAM-B3LYP}$	$E_{BHANDHLYP}$	ΔE_{B3LYP}	$\Delta E_{CAM-B3LYP}$	$\Delta E_{BHANDHLYP}$
pyrido[m,n]diazepines	[1,2]	N6 (N1-H)	-473.419641	-473.171227	-473.127105	4.96	5.02	5.11
		N7 (N1-H)	-473.420807	-473.172269	-473.128558	4.23	4.36	4.20
		N8 (N1-H)	-473.417710	-473.169223	-473.125086	6.18	6.28	6.38
		N9 (N1-H)	-473.427552	-473.179218	-473.135250	0.00	0.00	0.00
	[1,3]	N6 (N3-H)	-473.452077	-473.204180	-473.161695	4.47	4.70	4.65
		N7 (N3-H)	-473.451104	-473.202995	-473.160864	5.08	5.44	5.17
		N8 (N3-H)	-473.450125	-473.201984	-473.159727	5.70	6.08	5.89
		N9 (N1-H)	-473.459200	-473.211664	-473.169104	0.00	0.00	0.00
	[1,4]	N6 (N1-H)	-473.436405	-473.188698	-473.145465	6.91	7.02	7.27
		N7 (N1-H)	-473.439271	-473.191601	-473.149041	5.11	5.20	5.03
		N8 (N1-H)	-473.435263	-473.187361	-473.144093	7.63	7.86	8.13
		N9 (N1-H)	-473.447418	-473.199876	-473.157046	0.00	0.00	0.00
	[1,5]	N7 (N1-H)	-473.441778	-473.193738	-473.151037	6.13	6.26	6.09
		N6 (N5-H)	-473.451552	-473.203705	-473.160728	0.00	0.00	0.00
	[2,3]	N8 (N2-H)	-473.393320	-473.139363	-473.094474	18.12	21.33	21.97
		N9 (N2-H)	-473.393804	-473.139996	-473.093968	17.81	20.93	22.29
		N6 (N3-H)	-473.422189	-473.173327	-473.129460	0.00	0.00	0.00
		N7 (N3-H)	-473.419272	-473.170238	-473.126223	1.83	1.94	2.03
	[2,4]	N6 (N6-H)	-473.409555	-473.159539	-473.116171	0.00	0.00	0.00
		N7 (N7-H)	-473.409271	-473.158621	-473.114561	0.18	0.58	1.01

Table S4. The energies and energy differences ΔE (kcal/mol) referred to **the globally most stable isomer of pyrido[m,n]diazepines** (m=1, n=2-5; m=2, n=3,4) calculated using different functionals: B3LYP, CAM-B3LYP, BHandHLYP where PC denotes pyridine condensation type, DT is a given diazepine type, and T is tautomer type.

		PC (T)	E_{B3LYP}	$E_{CAM\text{-}B3LYP}$	$E_{BHANDHLYP}$	ΔE_{B3LYP}	$\Delta E_{CAM\text{-}B3LYP}$	$\Delta E_{BHANDHLYP}$
**pyrido[m,n]diazepines**	**[1,2]**	N6 (N1-H)	-473.419641	-473.171227	-473.127105	24.84	25.39	26.37
		N7 (N1-H)	-473.420807	-473.172269	-473.128558	24.11	24.74	25.46
		N8 (N1-H)	-473.417710	-473.169223	-473.125086	26.05	26.65	27.64
		N9 (N1-H)	-473.427552	-473.179218	-473.135250	19.87	20.37	21.26
	**[1,3]**	N6 (N3-H)	-473.452077	-473.204180	-473.161695	4.47	4.70	4.65
		N7 (N3-H)	-473.451104	-473.202995	-473.160864	5.08	5.44	5.17
		N8 (N3-H)	-473.450125	-473.201984	-473.159727	5.70	6.08	5.89
		N9 (N1-H)	**-473.459200**	**-473.211664**	**-473.169104**	0.00	0.00	0.00
	**[1,4]**	N6 (N1-H)	-473.436405	-473.188698	-473.145465	14.31	14.42	14.84
		N7 (N1-H)	-473.439271	-473.191601	-473.149041	12.51	12.60	12.60
		N8 (N1-H)	-473.435263	-473.187361	-473.144093	15.03	15.26	15.70
		N9 (N1-H)	-473.447418	-473.199876	-473.157046	7.40	7.40	7.57
	**[1,5]**	N7 (N1-H)	-473.441778	-473.193738	-473.151037	10.94	11.26	11.34
		N6 (N5-H)	-473.451552	-473.203705	-473.160728	4.80	5.00	5.26
		N8 (N2-H)	-473.393320	-473.139363	-473.094474	41.37	45.40	46.86
		N9 (N2-H)	-473.393804	-473.139996	-473.093968	41.06	45.00	47.18
	**[2,3]**	N6 (N3-H)	-473.422189	-473.173327	-473.129460	23.24	24.07	24.89
		N7 (N3-H)	-473.419272	-473.170238	-473.126223	25.07	26.01	26.93
	**[2,4]**	N6 (N6-H)	-473.409555	-473.159539	-473.116171	31.17	32.73	33.24
		N7 (N7-H)	-473.409271	-473.158621	-473.114561	31.35	33.31	34.25

Table S5. The energies and Gibbs free energy differences ΔG (kcal/mol) referred to **the most stable diazepine type isomers of pyrido[m,n]diazepines** (m=1, n=2-5; m=2, n=3,4) calculated using different functionals: B3LYP, CAM-B3LYP, BHandHLYP where PC denotes pyridine condensation type, DT is a given diazepine type, and T is tautomer type.

	PC	(T)	G_{B3LYP}	$G_{CAM-B3LYP}$	$G_{BHANDHLYP}$	ΔG_{B3LYP}	$\Delta G_{CAM-B3LYP}$	$\Delta G_{BHANDHLYP}$
pyrido[m,n]diazepines	[1,2]	N6 (N1-H)	-473.312429	-473.061766	-473.013838	4.90	4.96	5.07
		N7 (N1-H)	-473.313502	-473.062732	-473.015210	4.22	4.35	4.21
		N8 (N1-H)	-473.310392	-473.059663	-473.011708	6.17	6.28	6.40
		N9 (N1-H)	-473.320232	-473.069669	-473.021914	0.00	0.00	0.00
	[1,3]	N6 (N3-H)	-473.345550	-473.095304	-473.049267	3.86	4.20	4.06
		N7 (N3-H)	-473.345928	-473.094290	-473.049023	3.62	4.83	4.21
		N8 (N3-H)	-473.343989	-473.093202	-473.047516	4.84	5.51	5.16
		N9 (N1-H)	-473.351702	-473.101990	-473.055739	0.00	0.00	0.00
	[1,4]	N6 (N1-H)	-473.329775	-473.079793	-473.032812	6.63	6.77	7.04
		N7 (N1-H)	-473.332369	-473.082459	-473.036154	5.00	5.10	4.94
		N8 (N1-H)	-473.328464	-473.078314	-473.031289	7.45	7.70	7.99
		N9 (N1-H)	-473.340341	-473.090583	-473.044024	0.00	0.00	0.00
	[1,5]	N7 (N1-H)	-473.335026	-473.084753	-473.038400	5.92	6.08	5.91
		N6 (N5-H)	-473.344459	-473.094438	-473.047822	0.00	0.00	0.00
	[2,3]	N8 (N2-H)	-473.286276	-473.030315	-472.981812	18.24	21.30	21.86
		N9 (N2-H)	-473.286833	-473.031009	-472.981195	17.89	20.87	22.24
		N6 (N3-H)	-473.315340	-473.064263	-473.016641	0.00	0.00	0.00
		N7 (N3-H)	-473.312509	-473.061219	-473.013424	1.78	1.91	2.02
	[2,4]	N6 (N6-H)	-473.304151	-473.051738	-473.004704	0.05	0.00	0.00
		N7 (N7-H)	-473.304237	-473.051152	-473.003576	0.00	0.37	0.71

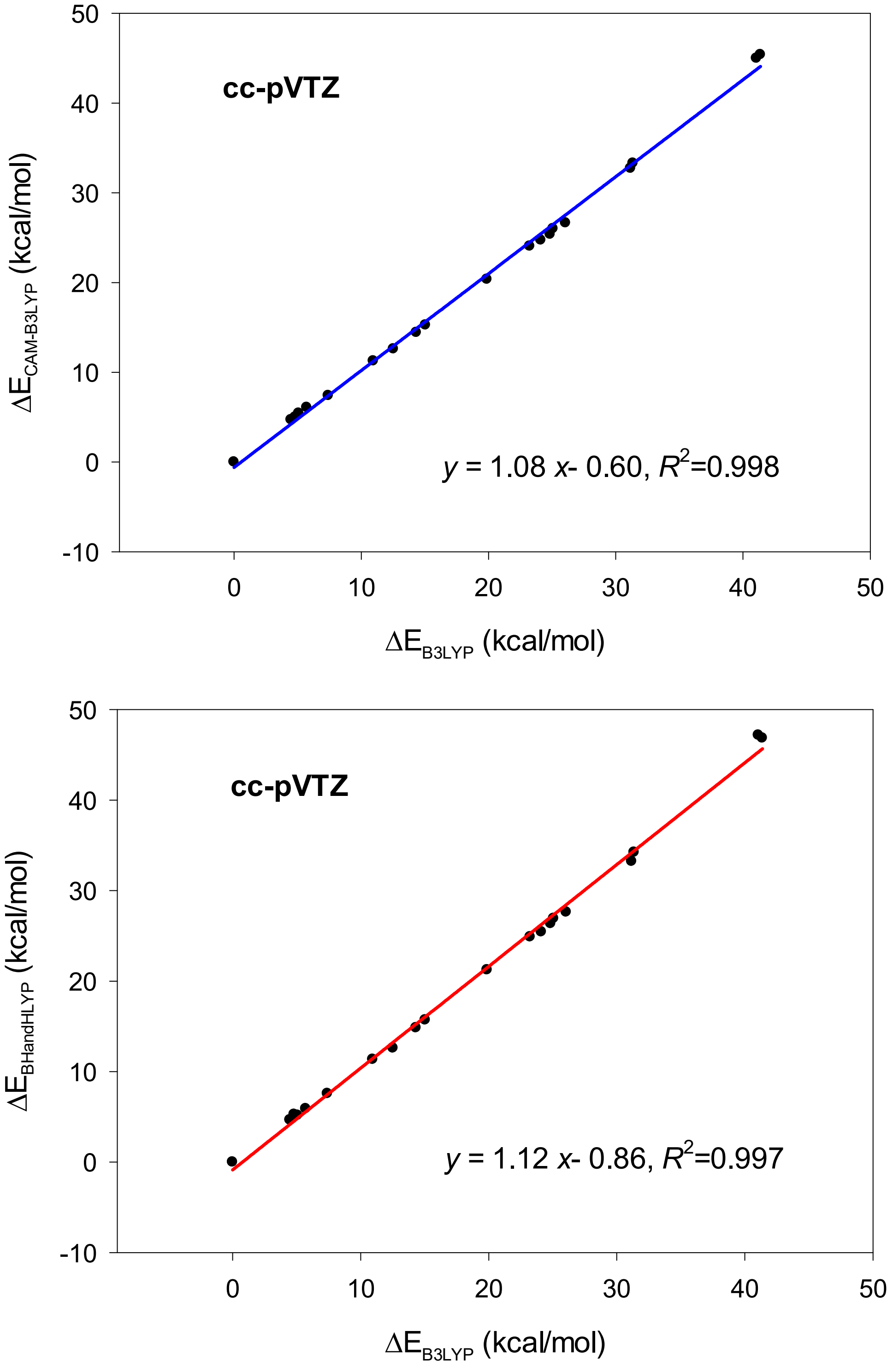

Figure S1. The correlations between energy differences ΔE (kcal/mol) of pyrido[m,n]diazepines (m=1, n=2-5; m=2, n=3,4) calculated using the B3LYP, CAM-B3LYP, and BHandHLYP functionals and the same cc-pVTZ basis set.

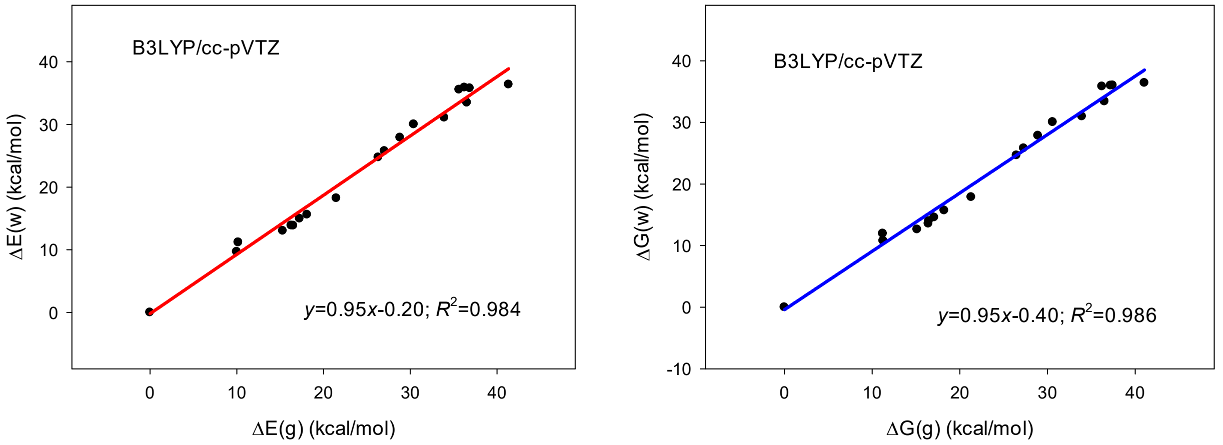

Figure S2. The correlations between relative (a) enery ΔE and (b) relative Gibbs fre energy ΔG (kcal/mol) of pyrido[m,n]diazepines (m=1, n=2-5; m=2, n=3,4) estimated using the B3LYP/cc-pVTZ method for molecules in the gas phase and water simulated using a PCM model.

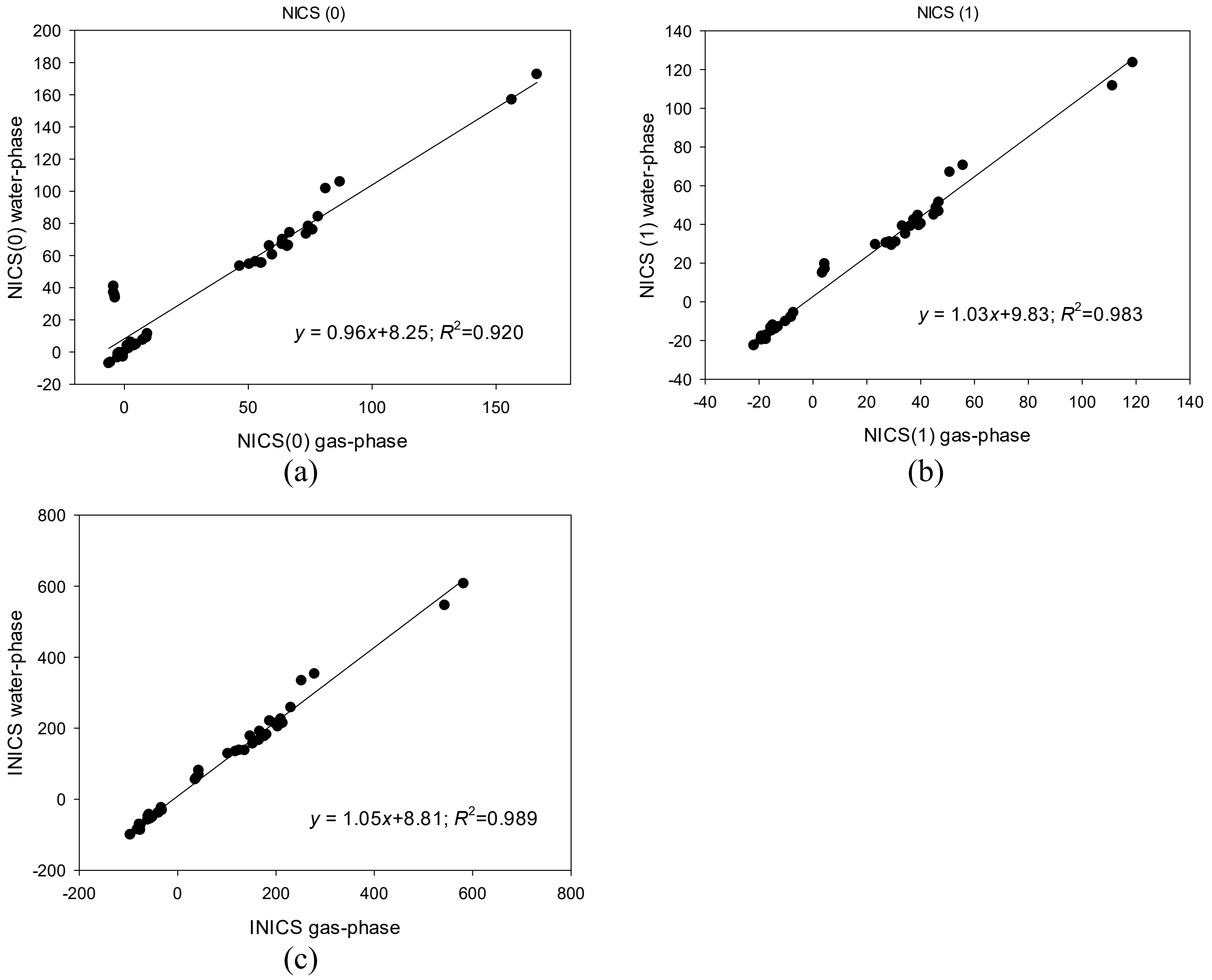

Figure S3. The correlations between NICS parameters of rings in pyrido[m,n]diazepines (m=1, n=2-5; m=2, n=3,4) estimated using the B3LYP/cc-pVTZ method for molecules in the gas phase and water simulated using a PCM model: (a) NICS(0); (b) NICS(1); and (c) INICS index.

PYRIDO[1,2]DIAZEPINES (GAS PHASE)

Pyridine ring (6)	Diazepine ring (7)
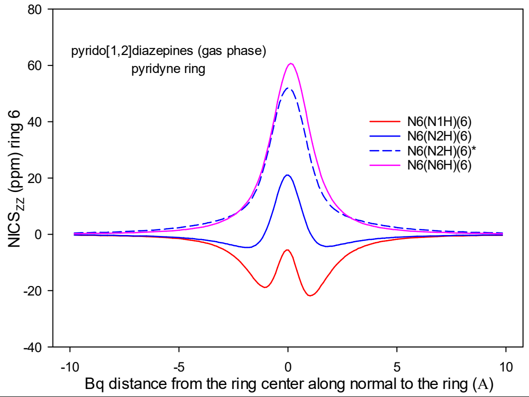	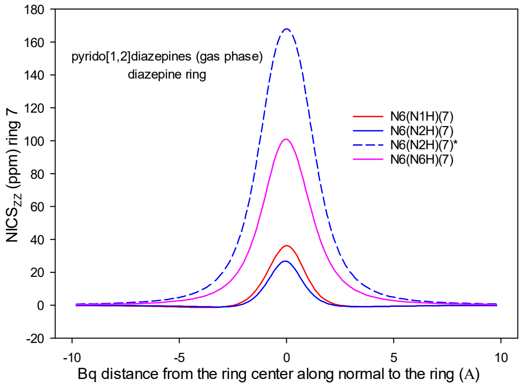
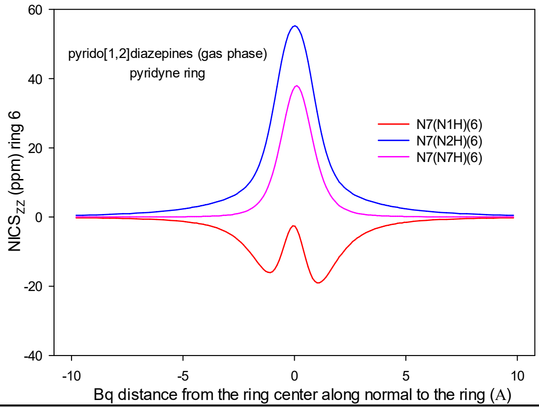	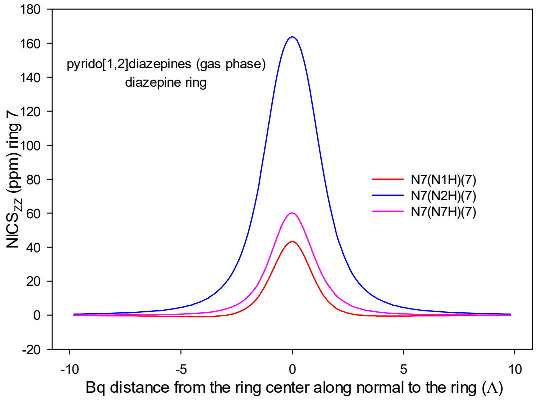
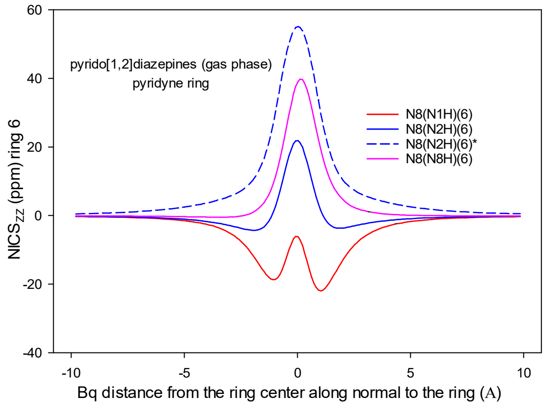	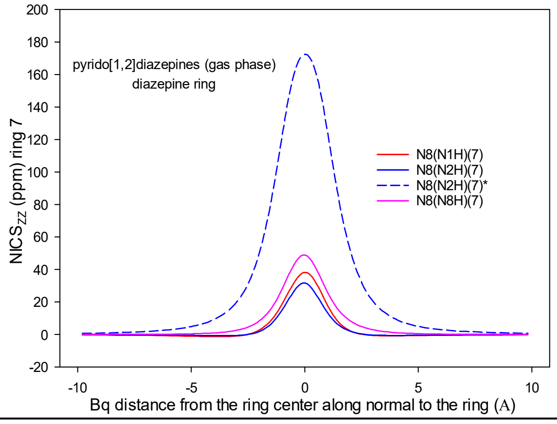
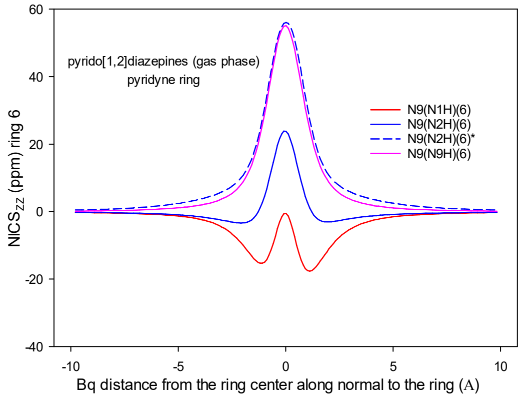	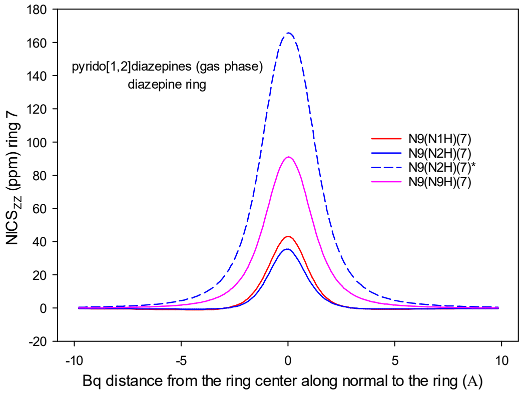

Figure S4. The NICSzz-scans vs. distance from the ring center along the normal to the ring for the pirydo[1,2]diazepines for N6, N7, N8, and N9 condensation type.

PYRIDO[1,3]DIAZEPINES (GAS PHASE)

Pyridine ring (6)	Diazepine ring (7)

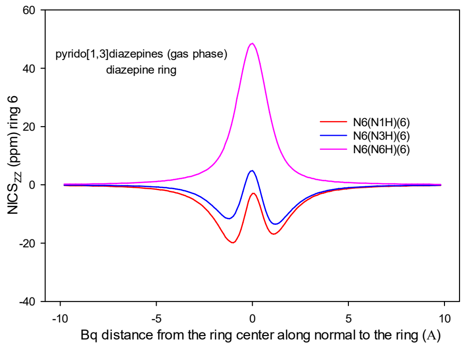

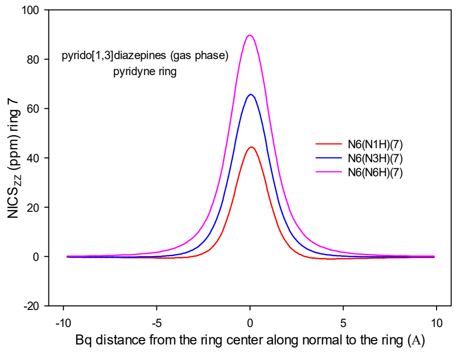

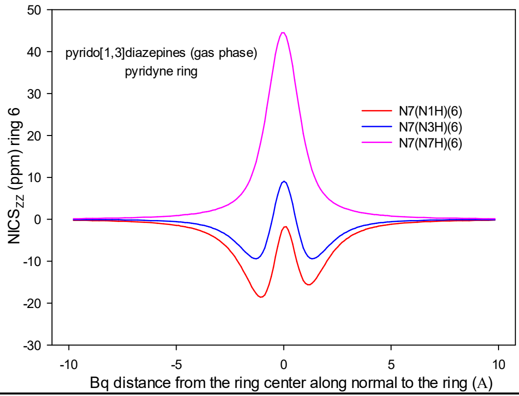

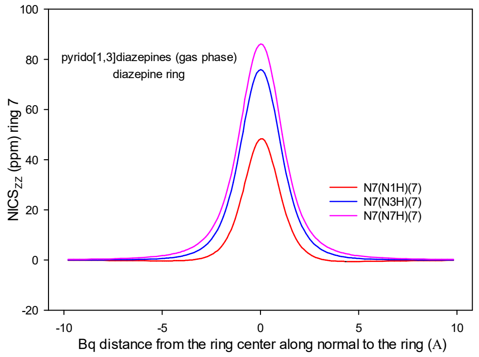

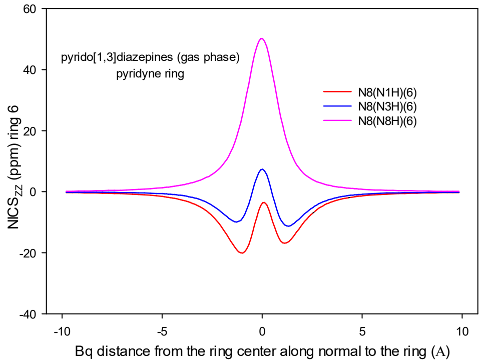

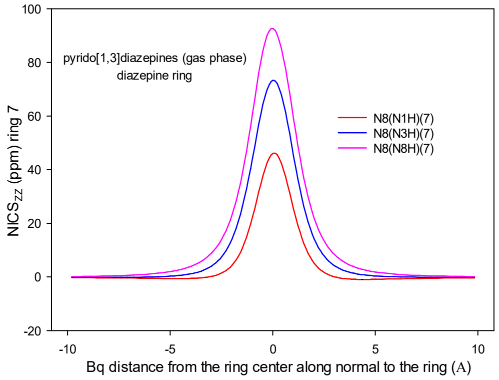

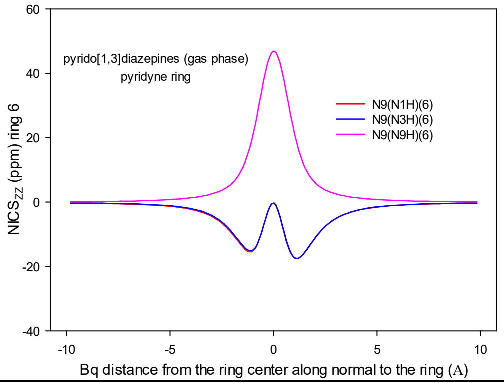

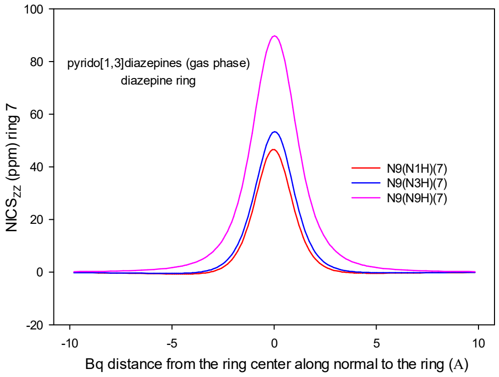

Figure S5. The NICSzz-scans vs. distance from the ring center along the normal to the ring for the pirydo[1,3]diazepines for N6, N7, N8, and N9 condensation type.

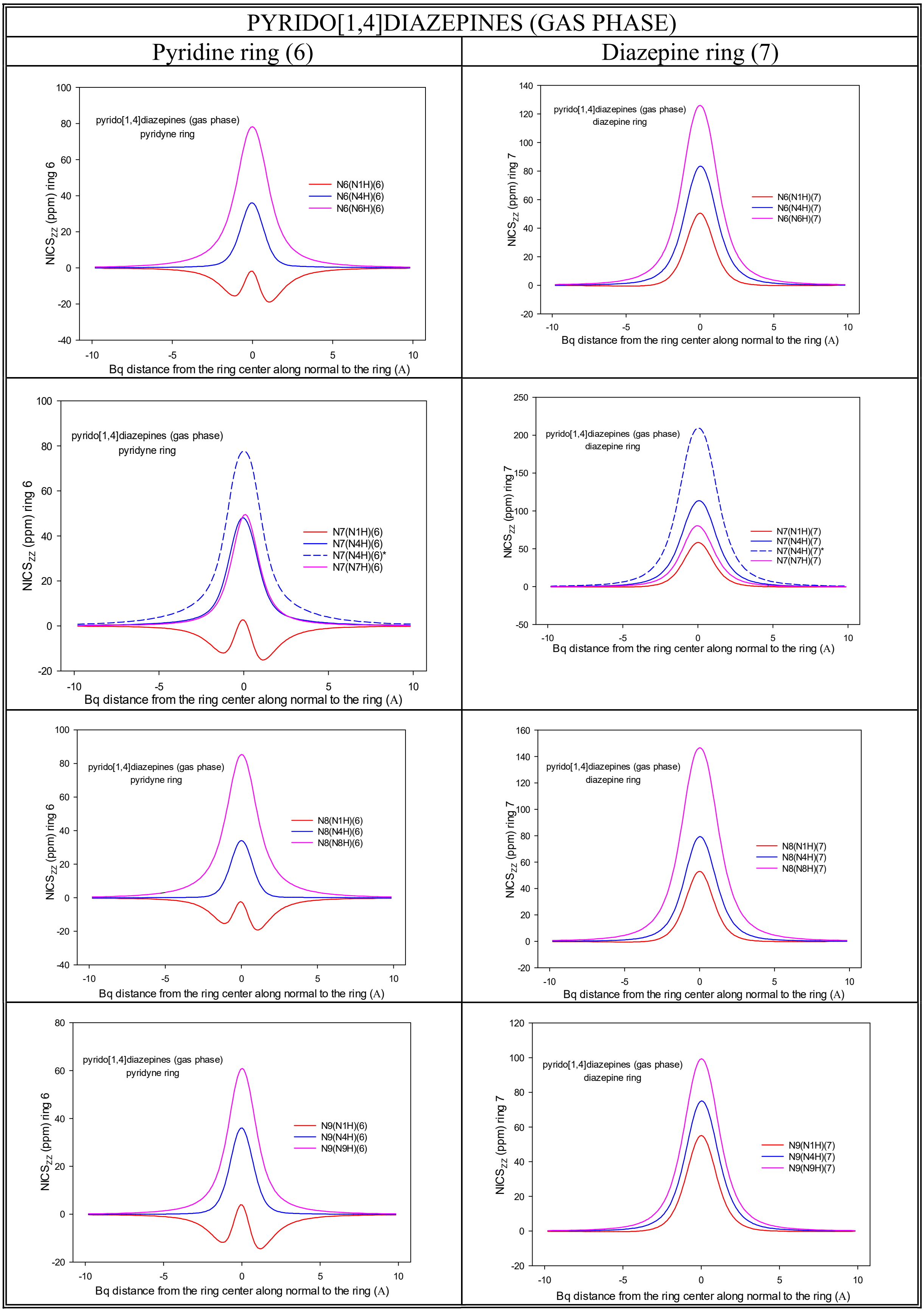

Figure S6. The NICSzz-scans vs. distance from the ring center along the normal to the ring for the pirydo[1,4]diazepines for N6, N7, N8, and N9 condensation type.

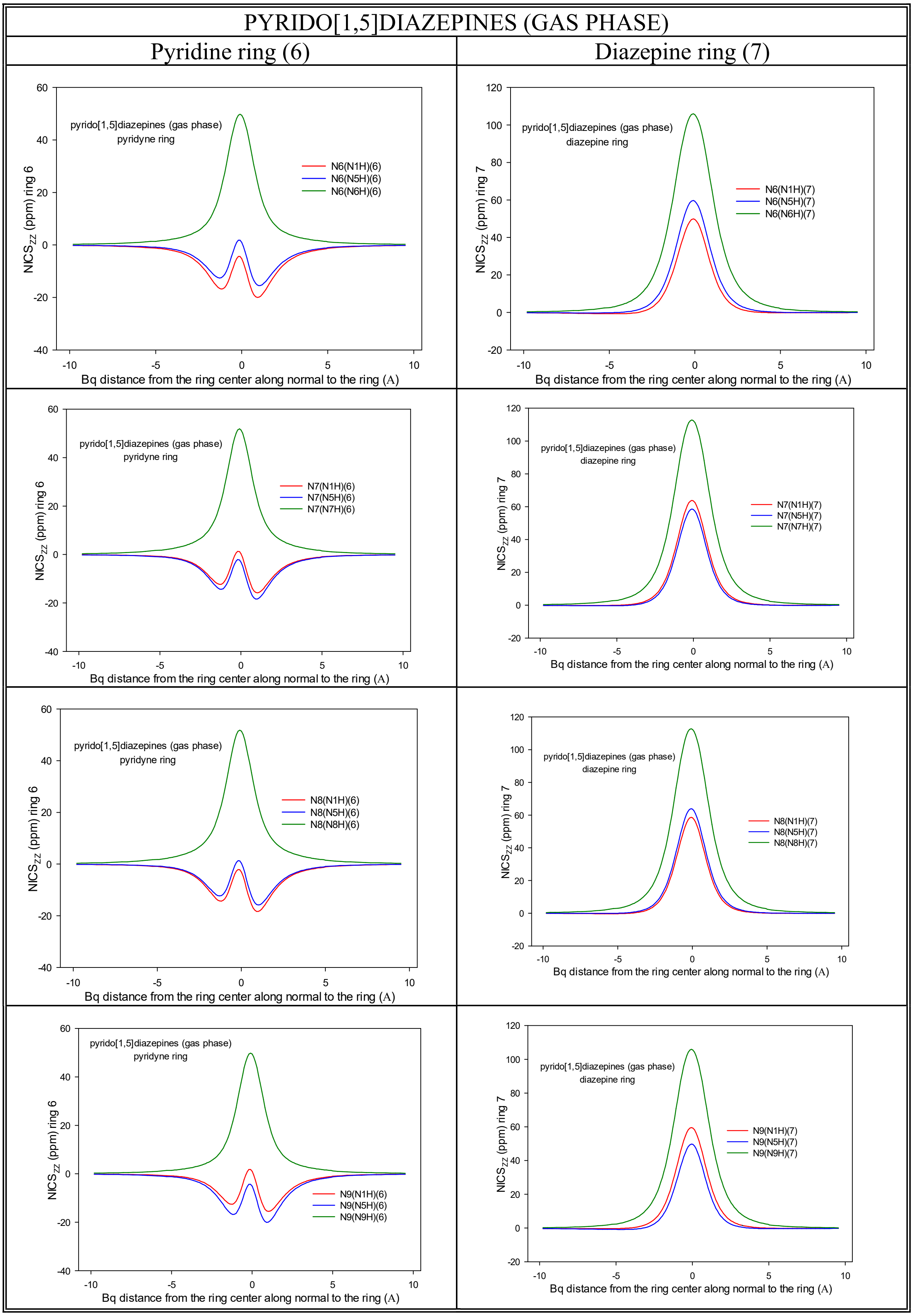

Figure S7. The NICSzz-scans vs. distance from the ring center along the normal to the ring for the pirydo[1,5]diazepines for N6, N7, N8, and N9 condensation type.

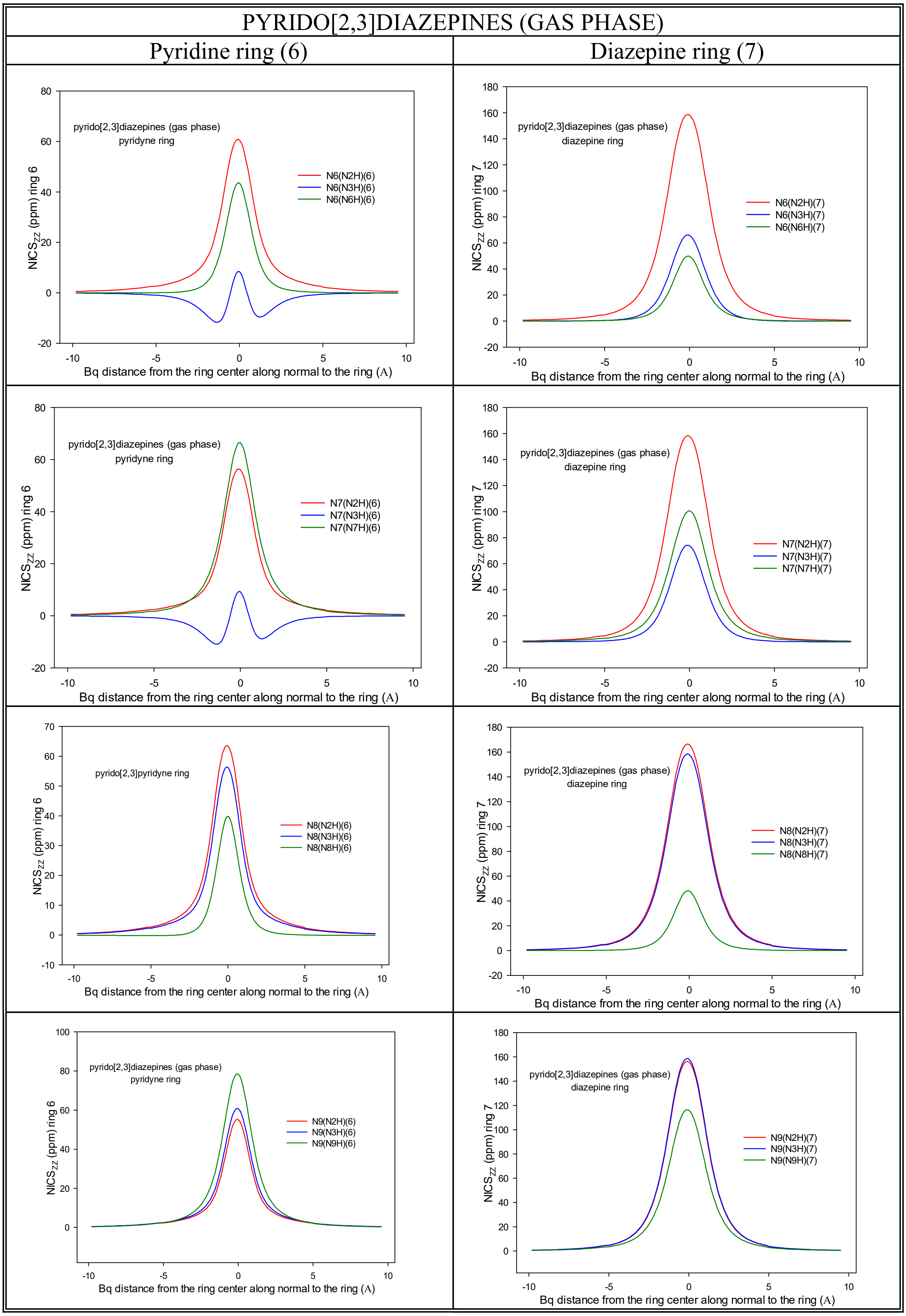

Figure S8. The NICSzz-scans vs. distance from the ring center along the normal to the ring for the pirydo[2,3]diazepines for N6, N7, N8, and N9 condensation type.

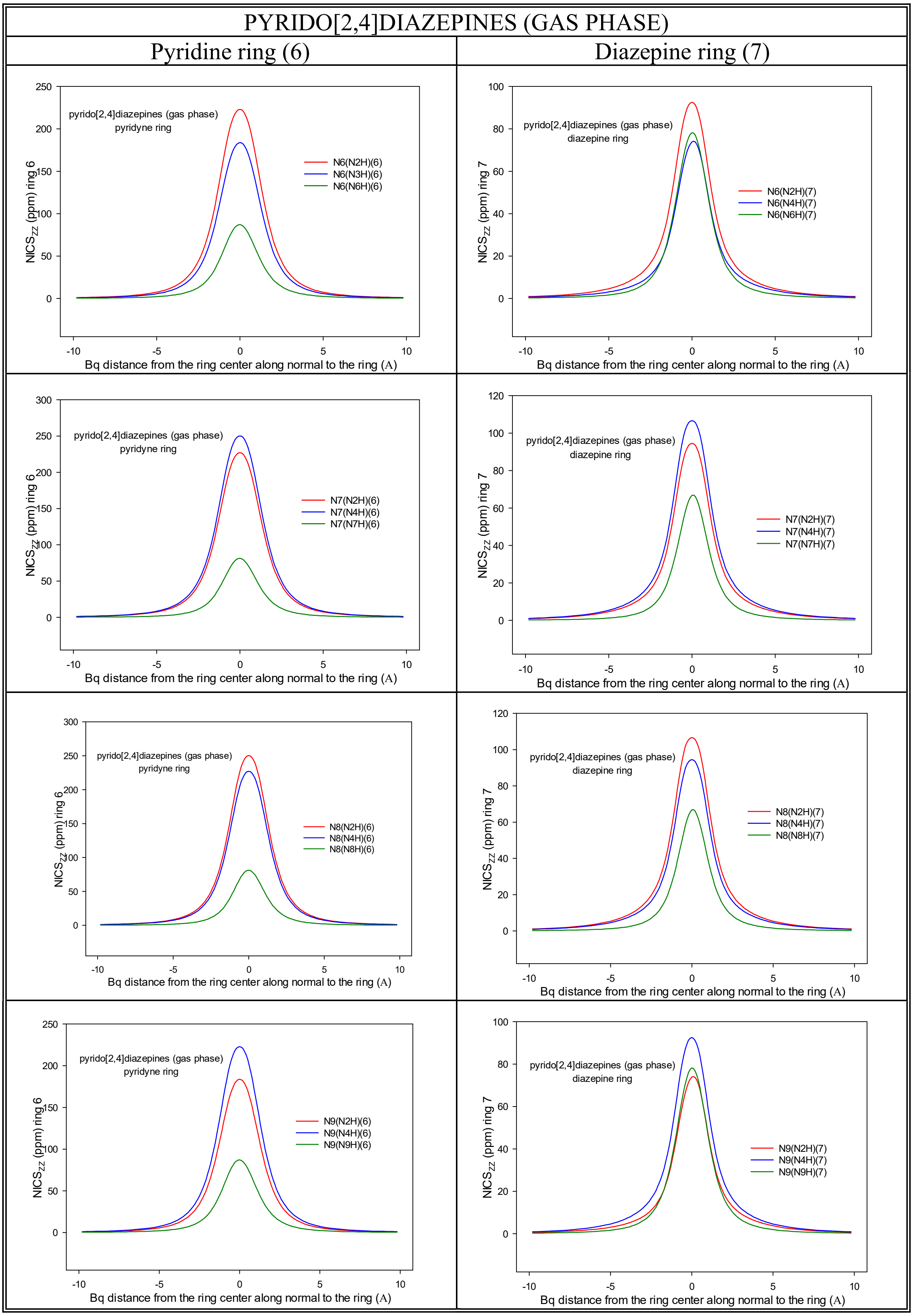

Figure S9. The NICSzz-scans vs. distance from the ring center along the normal to the ring for the pirydo[2,4]diazepines for N6, N7, N8, and N9 condensation type.

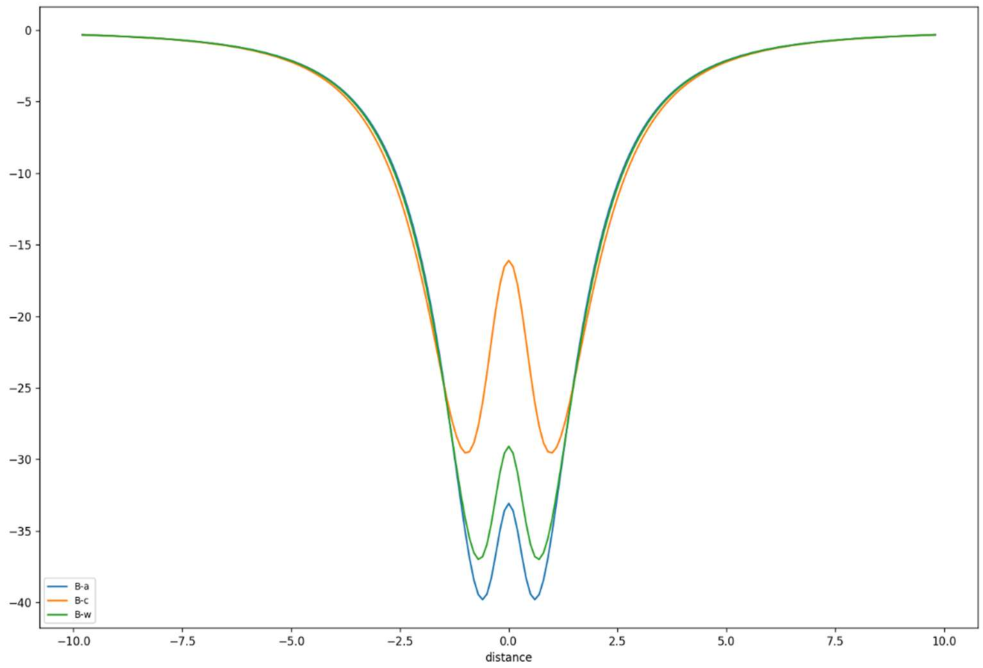

Figure S10. The NICS curves taken for the normal straight lines through the center of the benzene plane (c - orange), the point in the middle from the center to the bond center (a-blue), and in the middle from the center to the C-atom (w - green). The INICSs are equal to: -145.63, -170.96, and -165.77, respectively.

Table S6 Different NICS aromaticity indices of ring in pyrido[1,2]diazepines in the gas phase. DT – diazepine type, PC - pyridine condensation type, T – tautomer type, R -ring type, P – planarity (p- planar; n- non-planar), I – integral NICS (INICS. ppm/Å), I- – integral NICS for the distances below the plane (ppm/Å), I+ – integral NICS for the distances below the plane (ppm/Å), ΔI=I(-) – I(+)(ppm/Å); NICS(0), NICS(1), NICS(-1), and ΔNICS(-1,1)= NICS(-1) - NICS(1) (ppm), MIN1 and MIN2 are minima of the NICS function for distances below and above the ring plane, respectively, and NICS(MIN1) and NICS(MIN2) are the NICS function values in MIN1 and MIN2.

Gas Phase																
**DT**	**PC**	**T**	**R**	**P**	**I**	**I(-)**	**I(+)**	**ΔI**	**NICS (0)**	**NICS (-1)**	**NICS (1)**	**ΔNICS (-1,1)**	**MIN1**	**NICS (MIN1)**	**MIN2**	**NICS (MIN2)**
pyrido[1,2]diazepines	N6	N1-H	6	n	-95.9	-44.7	-51.2	-6.5	-5.6	-18.7	-21.8	3.1	-1.1	-18.8	1.0	-21.8
		N2-H	6	n	-0.4	0.1	-0.5	-0.6	21.1	1.9	0.5	1.4	-1.9	-4.7	1.8	-4.3
		N2-H	6	p	158.8	79.4	79.4	0.0	52.1	27.5	27.5	0.0	-9.8	0.5	9.8	0.5
		N6-H	6	n	77.0	43.7	33.2	-10.5	40.1	18.2	13.8	4.5	-7.7	0.0	3.7	-0.4
		N6-H	6	n	171.5	80.0	91.6	11.6	60.3	29.0	36.0	-6.9	-9.8	0.3	9.8	0.3
	N7	N1-H	6	n	-81.7	-37.3	-44.4	-7.1	-2.7	-15.8	-19.0	3.3	-1.1	-16.0	1.1	-19.0
		N2-H	6	p	168.6	84.3	84.3	0.0	55.3	30.5	30.5	0.0	-9.8	0.5	9.8	0.5
		N7-H	6	n	74.7	33.6	41.1	7.4	37.7	12.5	16.8	-4.3	-9.8	0.0	9.8	0.0
	N8	N1-H	6	n	-96.0	-44.4	-51.5	-7.1	-6.2	-18.6	-21.9	3.3	-1.1	-18.7	1.0	-21.9
		N2-H	6	n	4.8	2.2	2.6	0.4	21.9	2.7	1.6	1.1	-2.0	-4.3	1.8	-3.6
		N2-H	6	p	170.2	85.1	85.1	0.0	55.1	30.2	30.2	0.0	-9.8	0.5	9.8	0.5
		N8-H	6	n	75.7	29.2	46.5	17.4	39.2	11.4	19.5	-8.1	-3.4	-0.4	8.6	0.0
	N9	N1-H	6	n	-75.6	-34.9	-40.7	-5.8	-0.5	-14.9	-17.4	2.5	-1.2	-15.2	1.1	-17.5
		N2-H	6	n	12.9	7.3	5.7	-1.6	23.9	4.7	2.7	2.0	-2.1	-3.3	1.9	-3.0
		N2-H	6	p	173.3	86.6	86.6	0.0	56.1	30.9	30.9	0.0	-9.8	0.5	9.8	0.5
		N9-H	6	n	146.7	74.5	72.1	-2.4	55.1	28.9	27.5	1.4	-9.8	0.2	9.8	0.2
	N6	N1-H	7	n	64.5	31.9	32.6	0.7	36.2	17.7	16.6	1.1	-3.7	-1.5	3.6	-1.0
		N2-H	7	n	41.7	22.4	19.3	-3.0	26.6	12.3	9.8	2.5	-3.2	-1.1	3.4	-1.0
		N2-H	7	p	588.9	294.4	294.4	0.0	168.0	120.7	120.7	0.0	-9.8	0.6	9.8	0.6
		N6-H	7	n	122.7	60.1	62.6	2.5	47.1	25.0	27.1	-2.2	-9.8	0.0	9.8	0.0
		N6-H	7	n	329.4	164.9	164.5	-0.4	101.0	66.2	65.4	0.8	-9.8	0.4	9.8	0.4
	N7	N1-H	7	n	90.1	45.4	44.7	-0.7	43.2	22.8	21.4	1.4	-4.3	-0.9	4.4	-0.5
		N2-H	7	p	569.4	284.7	284.7	0.0	163.8	116.7	116.7	0.0	-9.8	0.6	9.8	0.6
		N7-H	7	n	163.5	81.1	82.3	1.2	60.1	34.2	34.2	0.0	-9.8	0.0	9.8	0.0
	N8	N1-H	7	n	70.5	35.3	35.2	-0.1	38.2	19.0	17.6	1.5	-3.8	-1.4	3.7	-0.9
		N2-H	7	n	57.5	29.9	27.6	-2.3	31.6	15.3	13.1	2.3	-3.6	-0.8	3.9	-0.7
		N2-H	7	p	604.5	302.2	302.2	0.0	172.6	123.8	123.8	0.0	-9.8	0.6	9.8	0.6
		N8-H	7	n	128.2	65.7	62.5	-3.1	48.8	28.4	25.6	2.8	-9.8	0.0	9.8	0.0
	N9	N1-H	7	n	92.1	46.6	45.5	-1.1	43.3	23.3	21.6	1.8	-4.3	-0.8	4.5	-0.5
		N2-H	7	n	74.3	38.6	35.7	-3.0	35.6	18.9	16.3	2.6	-4.3	-0.5	4.6	-0.4
		N2-H	7	p	580.5	290.2	290.2	0.0	165.9	118.8	118.8	0.0	-9.8	0.6	9.8	0.6
		N9-H	7	n	287.5	144.2	143.3	-0.9	91.2	58.9	58.9	0.0	-9.8	0.2	9.8	0.2

Table S7. Different NICS aromaticity indices of ring in pyrido[1,3]diazepines in the gas phase. DT – diazepine type, PC - pyridine condensation type, T – tautomer type, R -ring type, P – planarity (p- planar; n- non-planar). I – integral NICS (INICS. ppm/Å), I- – integral NICS for the distances below the plane (ppm/Å), I+ – integral NICS for the distances below the plane (ppm/Å), ΔI=I(-) – I(+)(ppm/Å); NICS(0), NICS(1), NICS(-1), and ΔNICS(-1,1)= NICS(-1) - NICS(1) (ppm), MIN1 and MIN2 are minima of the NICS function for distances below and above the ring plane, respectively, and NICS(MIN1) and NICS(MIN2) are the NICS function values in MIN1 and MIN2.

Gas Phase																
**DT**	**PC**	**T**	**R**	**P**	**I**	**I-**	**I+**	**ΔI**	**NICS (0)**	**NICS (-1)**	**NICS (1)**	**ΔNICS (-1,1)**	**MIN1**	**NICS (MIN1)**	**MIN2**	**NICS MIN2)**
**pyrido[1,3]diazepines**	N6	N1-H	6	n	-85.0	-46.0	-39.0	7.0	-3.0	-19.9	-16.7	-3.1	-1.0	-19.9	1.1	-17.0
		N3-H	6	n	-50.9	-22.6	-28.3	-5.7	4.8	-10.8	-13.0	2.2	-1.2	-11.6	1.2	-13.6
		N6-H	6	n	120.8	63.1	57.7	-5.5	48.5	23.4	21.9	1.5	-9.8	0.2	9.8	0.1
	N7	N1-H	6	n	-78.4	-42.9	-35.6	7.3	-1.9	-18.5	-15.3	-3.2	-1.1	-18.6	1.1	-15.6
		N3-H	6	p	-31.4	-15.7	-15.7	0.0	9.0	-8.0	-8.0	0.0	-1.3	-9.4	1.3	-9.4
		N7-H	6	n	106.0	55.6	50.5	-5.1	44.5	20.6	17.9	2.7	-9.8	0.2	9.8	0.1
	N8	N1-H	6	n	-85.3	-46.5	-38.9	7.6	-3.6	-20.0	-16.6	-3.4	-1.0	-20.0	1.1	-16.9
		N3-H	6	n	-38.3	-17.1	-21.1	-4.0	7.5	-8.5	-10.1	1.6	-1.3	-9.8	1.3	-11.2
		N8-H	6	n	129.1	69.9	59.2	-10.7	50.3	25.6	21.8	3.8	-9.8	0.2	9.8	0.2
	N9	N1-H	6	n	-74.8	-34.6	-40.2	-5.7	-0.3	-15.0	-17.3	2.3	-1.1	-15.3	1.1	-17.5
		N3-H	6	n	-73.0	-33.1	-39.8	-6.7	-0.2	-14.7	-17.2	2.5	-1.1	-15.0	1.1	-17.4
		N9-H	6	p	114.4	57.2	57.2	0.0	47.0	21.3	21.3	0.0	-9.8	0.2	9.8	0.2
	N6	N1-H	7	n	94.6	46.5	48.1	1.6	44.5	22.0	24.2	-2.2	-4.6	-0.5	4.3	-0.9
		N3-H	7	n	176.8	88.1	88.7	0.6	65.8	39.5	39.4	0.1	-7.4	-0.1	8.3	-0.1
		N6-H	7	p	280.9	143.1	137.8	-5.4	89.8	59.2	56.8	2.4	-9.8	0.2	9.8	0.2
	N7	N1-H	7	n	107.6	52.7	54.9	2.2	48.4	24.5	26.7	-2.3	-4.9	-0.4	4.7	-0.7
		N3-H	7	n	214.0	107.0	107.0	0.0	76.0	46.8	46.8	0.0	-9.8	0.0	9.8	0.0
		N7-H	7	n	265.1	133.1	132.0	-1.1	86.1	54.9	54.8	0.0	-9.8	0.2	9.8	0.2
	N8	N1-H	7	n	99.0	48.4	50.7	2.3	46.3	22.7	25.2	-2.5	-4.6	-0.5	4.4	-0.8
		N3-H	7	n	204.0	101.7	102.3	0.6	73.4	44.7	44.9	-0.2	-9.5	0.0	9.8	0.0
		N8-H	7	n	293.0	149.7	143.4	-6.3	92.7	61.4	58.8	2.6	-9.8	0.3	9.8	0.2
	N9	N1-H	7	n	102.5	52.6	49.9	-2.7	46.6	25.9	23.2	2.7	-4.6	-0.7	4.8	-0.4
		N3-H	7	n	128.1	63.7	64.4	0.7	53.4	30.0	29.6	0.5	-5.3	-0.5	5.9	-0.2
		N9-H	7	p	278.3	139.2	139.2	0.0	89.9	57.7	57.7	0.0	-9.8	0.2	9.8	0.2

Table S8. Different NICS aromaticity indices of ring in pyrido[1,4]diazepines in the gas phase. DT – diazepine type, PC - pyridine condensation type, T – tautomer type, R -ring type, P – planarity (p- planar; n- non-planar), I – integral NICS (INICS, ppm/Å), I- – integral NICS for the distances below the plane (ppm/Å), I+ – integral NICS for the distances below the plane (ppm/Å), ΔI=I(-) – I(+)(ppm/Å); NICS(0), NICS(1), NICS(-1), and ΔNICS(-1,1)= NICS(-1) - NICS(1) (ppm), MIN1 and MIN2 are minima of the NICS function for distances below and above the ring plane, respectively, and NICS(MIN1) and NICS(MIN2) are the NICS function values in MIN1 and MIN2.

Gas Phase																	
**DT**	**PC**	**T**	**R**	**P**	**I**	**I-**	**I+**	**ΔI**	**NICS (0)**	**NICS (-1)**	**NICS (1)**	**ΔNICS (-1,1)**	**MIN1**	**NICS (MIN1)**	**MIN2**	**NICS (MIN2)**
**pyrido[1,4]diazepines**	N6	N1-H	6	n	-77.5	-34.9	-42.6	-7.7	-1.9	-15.3	-18.9	3.6	-1.1	-15.6	1.0	-18.9
		N4-H	6	n	73.4	38.2	35.2	-3.0	36.1	14.9	12.9	2.0	-9.8	0.1	9.8	0.1
		N6-H	6	n	243.2	124.5	118.7	-5.8	78.3	47.0	45.8	1.2	-9.8	0.5	9.8	0.4
	N7	N1-H	6	n	-57.7	-25.0	-32.7	-7.7	2.6	-11.2	-14.8	3.6	-1.2	-12.0	1.1	-15.1
		N4-H	6	n	128.8	65.8	63.1	-2.7	48.0	25.7	23.4	2.3	-9.8	0.2	9.8	0.3
		N4-H	6	p	266.1	133.1	133.1	0.0	77.7	48.8	48.8	0.0	-9.8	0.8	9.8	0.8
		N7-H	6	n	122.6	57.2	65.4	8.1	49.3	21.1	25.4	-4.3	-9.8	0.1	9.8	0.1
	N8	N1-H	6	n	-77.3	-34.2	-43.1	-8.8	-2.5	-14.9	-19.1	4.1	-1.1	-15.2	1.0	-19.1
		N4-H	6	n	65.0	33.2	31.8	-1.4	34.2	13.3	12.2	1.0	-9.8	0.0	9.8	0.0
		N8-H	6	p	278.3	139.1	139.2	0.0	85.5	51.7	51.7	0.0	-9.8	0.6	9.8	0.6
	N9	N1-H	6	n	-55.0	-24.0	-30.9	-6.9	4.0	-10.8	-13.9	3.1	-1.2	-11.7	1.2	-14.3
		N4-H	6	n	71.4	36.7	34.7	-2.0	36.1	15.0	13.6	1.4	-9.8	0.0	9.8	0.0
		N9-H	6	p	170.3	85.1	85.1	0.0	60.9	32.7	32.7	0.0	-9.8	0.3	9.8	0.3
	N6	N1-H	7	n	117.9	58.2	59.7	1.5	50.5	28.1	27.2	0.9	-4.8	-0.6	5.9	-0.2
		N4-H	7	n	255.0	124.5	130.5	6.0	83.5	52.2	55.0	-2.7	-9.8	0.1	9.8	0.2
		N6-H	7	n	431.0	217.5	213.5	-3.9	126.1	87.3	86.4	0.9	-9.8	0.5	9.8	0.5
	N7	N1-H	7	n	147.8	73.3	74.5	1.3	58.6	33.7	33.2	0.4	-5.9	-0.3	7.4	-0.1
		N4-H	7	n	371.9	182.4	189.5	7.1	113.8	75.1	78.7	-3.5	-9.8	0.3	9.8	0.3
		N4-H	7	p	751.5	375.7	375.7	0.0	209.7	152.5	152.5	0.0	-9.8	0.9	9.8	0.9
		N7-H	7	n	246.4	125.2	121.2	-4.0	80.5	52.3	49.4	2.9	-9.8	0.2	9.8	0.2
	N8	N1-H	7	n	125.3	62.2	63.0	0.8	53.0	29.7	28.5	1.2	-5.0	-0.5	5.9	-0.2
		N4-H	7	n	236.1	115.8	120.3	4.5	79.4	48.9	50.8	-1.9	-9.8	0.1	9.8	0.1
		N8-H	7	p	514.2	257.1	257.1	0.0	146.8	103.3	103.3	0.0	-9.8	0.7	9.8	0.7
	N9	N1-H	7	n	136.6	67.9	68.7	0.8	55.1	31.6	30.8	0.8	-5.6	-0.3	6.8	-0.1
		N4-H	7	n	221.7	108.7	113.0	4.3	75.1	46.0	47.9	-1.9	-9.8	0.1	9.8	0.1
		N9-H	7	p	320.9	160.4	160.4	0.0	99.4	65.6	65.6	0.0	-9.8	0.3	9.8	0.3

Table S9. Different NICS aromaticity indices of ring in pyrido[1,5]diazepines in the gas phase. DT – diazepine type, PC - pyridine condensation type, T – tautomer type, R -ring type, P – planarity (p- planar; n- non-planar), I – integral NICS (INICS. ppm/Å), I- – integral NICS for the distances below the plane (ppm/Å), I+ – integral NICS for the distances below the plane (ppm/Å), ΔI=I(-) – I(+)(ppm/Å); NICS(0), NICS(1), NICS(-1), and ΔNICS(-1,1)= NICS(-1) - NICS(1) (ppm), MIN1 and MIN2 are minima of the NICS function for distances below and above the ring plane, respectively, and NICS(MIN1) and NICS(MIN2) are the NICS function values in MIN1 and MIN2.

DT	PC	T	R	P	I	I-	I+	ΔI	NICS (0)	NICS (-1)	NICS (1)	ΔNICS (-1,1)	MIN1	NICS (MIN1)	MIN2	NICS (MIN2)
Gas Phase																
pyrido[1,5]diazepines	N6	N1-H	6	n	-84.5	-38.1	-46.4	-8.3	-4.4	-16.7	-20.0	3.3	-1.1	-16.8	1.0	-20.0
		N5-H	6	n	-60.3	-26.0	-34.3	-8.2	1.8	-12.1	-15.3	3.2	-1.2	-12.6	1.1	-15.5
		N6-H	6	p	133.1	66.6	66.6	0.0	49.9	24.4	24.4	0.0	-9.8	0.2	9.8	0.2
	N7	N1-H	6	n	-60.0	-25.4	-34.6	-9.3	1.2	-11.8	-15.6	3.8	-1.2	-12.4	1.1	-15.8
		N5-H	6	n	-72.9	-31.4	-41.6	-10.2	-2.2	-14.1	-18.4	4.2	-1.1	-14.4	1.1	-18.4
		N7-H	6	p	144.4	72.2	72.2	0.0	51.9	25.6	25.6	0.0	-9.8	0.3	9.8	0.3
	N8	N1-H	6	n	-72.9	-31.4	-41.6	-10.2	-2.2	-14.1	-18.4	4.2	-1.1	-14.4	1.1	-18.4
		N5-H	6	n	-60.0	-25.4	-34.6	-9.3	1.2	-11.8	-15.6	3.8	-1.2	-12.4	1.1	-15.8
		N8-H	6	p	144.4	72.2	72.2	0.0	51.9	25.6	25.6	0.0	-9.8	0.3	9.8	0.3
	N9	N1-H	6	n	-60.3	-26.0	-34.3	-8.2	1.8	-12.1	-15.3	3.2	-1.2	-12.6	1.1	-15.5
		N5-H	6	n	-84.5	-38.1	-46.4	-8.3	-4.4	-16.7	-20.0	3.3	-1.1	-16.8	1.0	-20.0
		N9-H	6	p	133.1	66.6	66.6	0.0	49.9	24.4	24.4	0.0	-9.8	0.2	9.8	0.2
	N6	N1-H	7	n	114.1	55.6	58.5	3.0	49.9	27.1	26.9	0.2	-4.6	-0.7	5.6	-0.2
		N5-H	7	n	153.2	75.9	77.3	1.4	59.7	35.0	34.4	0.6	-5.9	-0.3	7.7	-0.1
		N6-H	7	p	343.9	171.9	171.9	0.0	106.0	70.9	70.9	0.0	-9.8	0.3	9.8	0.3
	N7	N1-H	7	n	167.0	82.5	84.5	2.0	63.9	37.5	37.4	0.1	-6.5	-0.2	8.3	-0.1
		N5-H	7	n	146.1	71.8	74.4	2.6	58.6	33.4	33.3	0.0	-5.6	-0.4	7.1	-0.1
		N7-H	7	p	373.5	186.8	186.8	0.0	112.9	76.6	76.6	0.0	-9.8	0.4	9.8	0.4
	N8	N1-H	7	n	146.1	71.8	74.4	2.6	58.6	33.4	33.3	0.0	-5.6	-0.4	7.1	-0.1
		N5-H	7	n	167.0	82.5	84.5	2.0	63.9	37.5	37.4	0.1	-6.5	-0.2	8.3	-0.1
		N8-H	7	p	373.5	186.8	186.8	0.0	112.9	76.6	76.6	0.0	-9.8	0.4	9.8	0.4
	N9	N1-H	7	n	153.2	75.9	77.3	1.4	59.7	35.0	34.4	0.6	-5.9	-0.3	7.7	-0.1
		N5-H	7	n	114.1	55.6	58.5	3.0	49.9	27.1	26.9	0.2	-4.6	-0.7	5.6	-0.2
		N9-H	7	p	343.9	171.9	171.9	0.0	106.0	70.9	70.9	0.0	-9.8	0.3	9.8	0.3

Table S10. Different NICS aromaticity indices of ring in pyrido[2,3]diazepines in the gas phase. DT – diazepine type, PC - pyridine condensation type, T – tautomer type, R -ring type, P – planarity (p- planar; n- non-planar), I – integral NICS (INICS ppm/Å), I- – integral NICS for the distances below the plane (ppm/Å), I+ – integral NICS for the distances below the plane (ppm/Å), ΔI=I(-) – I(+)(ppm/Å); NICS(0), NICS(1), NICS(-1), and ΔNICS(-1,1)= NICS(-1) - NICS(1) (ppm), MIN1 and MIN2 are minima of the NICS function for distances below and above the ring plane, respectively, and NICS(MIN1) and NICS(MIN2) are the NICS function values in MIN1 and MIN2.

Gas Phase																
**DT**	**PC**	**T**	**R**	**P**	**I**	**I-**	**I+**	**ΔI**	**NICS (0)**	**NICS (-1)**	**NICS (1)**	**ΔNICS (-1.1)**	**MIN1**	**NICS (MIN1)**	**MIN2**	**NICS (MIN2)**
**pyrido[2,3]diazepines**	N6	N2-H	6	p	186.9	93.5	93.5	0.0	61.0	33.8	33.8	0.0	-9.8	0.5	9.8	0.5
		N3-H	6	n	-38.9	-22.4	-16.4	6.0	8.4	-10.8	-8.2	-2.6	-1.2	-11.7	1.3	-9.6
		N6-H	6	n	91.0	43.2	47.7	4.5	43.6	17.6	19.5	-1.9	-7.1	0.0	9.8	0.0
	N7	N2-H	6	p	168.4	84.2	84.2	0.0	56.4	30.3	30.3	0.0	-9.8	0.5	9.8	0.5
		N3-H	6	n	-32.8	-19.4	-13.4	5.9	9.3	-9.7	-7.2	-2.5	-1.3	-10.9	1.3	-8.8
		N7-H	6	n	191.8	91.5	100.3	8.7	66.5	34.5	38.1	-3.6	-9.8	0.3	9.8	0.3
	N8	N2-H	6	p	198.2	99.1	99.1	0.0	63.6	36.2	36.2	0.0	-9.8	0.5	9.8	0.5
		N3-H	6	p	168.4	84.2	84.2	0.0	56.4	30.3	30.3	0.0	-9.8	0.5	9.8	0.5
		N8-H	6	n	77.0	34.5	42.5	7.9	39.8	13.6	17.3	-3.7	-4.7	-0.1	9.2	0.0
	N9	N2-H	6	n	165.6	82.8	82.8	0.0	55.4	29.3	29.3	0.0	-9.8	0.5	9.8	0.5
		N3-H	6	p	186.9	93.5	93.5	0.0	61.0	33.8	33.8	0.0	-9.8	0.5	9.8	0.5
		N9-H	6	p	240.5	120.3	120.3	0.0	78.7	46.0	46.0	0.0	-9.8	0.4	9.8	0.4
	N6	N2-H	7	p	554.0	277.0	277.0	0.0	158.7	113.2	113.2	0.0	-9.8	0.6	9.8	0.6
		N3-H	7	n	181.2	90.9	90.3	-0.5	66.2	40.0	40.2	-0.2	-9.8	0.0	8.0	-0.1
		N6-H	7	n	127.9	62.0	65.9	3.9	50.0	26.1	27.5	-1.4	-9.8	0.0	9.8	0.0
	N7	N2-H	7	p	549.5	274.7	274.7	0.0	158.5	112.5	112.5	0.0	-9.8	0.6	9.8	0.6
		N3-H	7	n	210.2	105.5	104.7	-0.8	74.3	46.0	45.8	0.2	-9.8	0.0	9.8	0.0
		N7-H	7	n	327.3	157.0	170.4	13.4	100.3	62.9	69.0	-6.1	-9.8	0.3	9.8	0.4
	N8	N2-H	7	p	582.1	291.1	291.1	0.0	166.5	118.8	118.8	0.0	-9.8	0.6	9.8	0.6
		N3-H	7	p	549.5	274.7	274.7	0.0	158.5	112.5	112.5	0.0	-9.8	0.6	9.8	0.6
		N8-H	7	n	118.8	57.7	61.1	3.4	48.2	24.4	25.5	-1.1	-9.8	0.0	9.8	0.0
	N9	N2-H	7	n	543.7	271.9	271.9	0.0	156.4	111.3	111.3	0.0	-9.8	0.6	9.8	0.6
		N3-H	6	p	554.0	277.0	277.0	0.0	158.7	113.2	113.2	0.0	-9.8	0.6	9.8	0.6
		N9-H	7	p	392.9	196.5	196.5	0.0	116.5	79.1	79.1	0.0	-9.8	0.5	9.8	0.5

Table S11. Different NICS aromaticity indices of ring in pyrido[2,4]diazepines in the gas phase. DT – diazepine type, PC - pyridine condensation type, T – tautomer type, R -ring type, P – planarity (p- planar; n- non-planar), I – integral NICS (INICS. ppm/Å), I- – integral NICS for the distances below the plane (ppm/Å), I+ – integral NICS for the distances below the plane (ppm/Å), ΔI=I(-) – I(+)(ppm/Å); NICS(0), NICS(1), NICS(-1), and ΔNICS(-1,1)= NICS(-1) - NICS(1) (ppm). MIN1 and MIN2 are minima of the NICS function for distances below and above the ring plane, respectively, and NICS(MIN1) and NICS(MIN2) are the NICS function values in MIN1 and MIN2.

Gas Phase																
**DT**	**PC**	**T**	**R**	**P**	**I**	**I-**	**I+**	**ΔI**	**NICS (0)**	**NICS (-1)**	**NICS (1)**	**ΔNICS (-1,1)**	**MIN1**	**NICS (MIN1)**	**MIN2**	**NICS (MIN2)**
pyrido[2,4]diazepines	N6	N2-H	6	p	808.0	404.0	404.0	0.0	223.1	162.8	162.8	0.0	-9.8	1.0	9.8	1.0
		N4-H	6	n	651.9	324.6	327.3	2.6	183.9	131.5	132.2	-0.7	-9.8	0.8	9.8	0.8
		N6-H	6	p	278.7	137.7	141.0	3.3	87.1	55.7	55.8	-0.1	-9.8	0.3	9.8	0.3
	N7	N2-H	6	p	820.6	410.3	410.3	0.0	227.2	165.5	165.5	0.0	-9.8	1.1	9.8	1.1
		N4-H	6	p	913.7	456.9	456.9	0.0	250.3	183.6	183.6	0.0	-9.8	1.2	9.8	1.2
		N7-H	6	n	252.1	124.4	127.7	3.3	81.3	50.6	50.9	-0.3	-9.8	0.2	9.8	0.3
	N8	N2-H	6	p	913.7	456.9	456.9	0.0	250.3	183.6	183.6	0.0	-9.8	1.2	9.8	1.2
		N4-H	6	p	820.6	410.3	410.3	0.0	227.2	165.5	165.5	0.0	-9.8	1.1	9.8	1.1
		N8-H	6	n	252.1	124.4	127.7	3.3	81.3	50.6	50.9	-0.3	-9.8	0.2	9.8	0.3
	N9	N2-H	6	n	651.9	324.6	327.3	2.6	183.9	131.5	132.2	-0.7	-9.8	0.8	9.8	0.8
		N4-H	6	p	807.9	404.0	404.0	0.0	223.0	162.8	162.8	0.0	-9.8	1.0	9.8	1.0
		N9-H	6	p	278.7	137.7	141.0	3.3	87.1	55.7	55.8	-0.1	-9.8	0.3	9.8	0.3
	N6	N2-H	7	p	325.1	162.5	162.5	0.0	92.5	59.8	59.8	0.0	-9.8	0.9	9.8	0.9
		N4-H	7	n	246.4	116.7	129.7	13.0	73.9	42.6	47.6	-5.1	-9.8	0.6	9.8	0.7
		N6-H	7	p	230.5	112.7	117.8	5.1	78.2	45.0	46.8	-1.8	-9.8	0.3	9.8	0.3
	N7	N2-H	7	p	331.3	165.6	165.6	0.0	94.5	61.8	61.8	0.0	-9.8	0.9	9.8	0.9
		N4-H	7	p	383.9	192.0	192.0	0.0	106.7	71.6	71.6	0.0	-9.8	1.1	9.8	1.1
		N7-H	7	n	187.4	88.8	98.5	9.7	66.8	34.6	39.0	-4.5	-9.8	0.2	9.8	0.2
	N8	N2-H	7	p	383.9	192.0	192.0	0.0	106.7	71.6	71.6	0.0	-9.8	1.1	9.8	1.1
		N4-H	7	p	331.3	165.6	165.6	0.0	94.5	61.8	61.8	0.0	-9.8	0.9	9.8	0.9
		N8-H	7	n	187.4	88.8	98.5	9.7	66.8	34.6	39.0	-4.5	-9.8	0.2	9.8	0.2
	N9	N2-H	7	n	246.4	116.7	129.7	13.0	73.9	42.6	47.6	-5.1	-9.8	0.6	9.8	0.7
		N4-H	7	p	325.2	162.6	162.6	0.0	92.6	59.9	59.9	0.0	-9.8	0.9	9.8	0.9
		N9-H	7	p	230.5	112.7	117.8	5.1	78.2	45.0	46.8	-1.8	-9.8	0.3	9.8	0.3

Table S12. Different NICS aromaticity indices of ring in pyrido[1,2]diazepines in the water phase. DT – diazepine type, PC - pyridine condensation type, T – tautomer type, R -ring type, P – planarity (p- planar; n- non-planar), I – integral NICS (INICS. ppm/Å), I- – integral NICS for the distances below the plane (ppm/Å), I+ – integral NICS for the distances below the plane (ppm/Å), ΔI=I(-) – I(+)(ppm/Å); NICS(0), NICS(1), NICS(-1), and ΔNICS(-1,1)= NICS(-1) - NICS(1) (ppm). MIN1 and MIN2 are minima of the NICS function for distances below and above the ring plane, respectively, and NICS(MIN1) and NICS(MIN2) are the NICS function values in MIN1 and MIN2.

Water Phase																
**DT**	**PC**	**T**	**R**	**P**	**I**	**I-**	**I+**	**ΔI**	**NICS (0)**	**NICS (-1)**	**NICS (1)**	**ΔNICS (-1,1)**	**MIN1**	**NICS (MIN1)**	**MIN2**	**NICS (MIN2)**
**pyrido[1,2]diazepines**	N6	N1-H	6	n	-100.0	-46.8	-53.1	-6.3	-6.4	-19.4	-22.5	3.1	-1.1	-19.5	1.0	-22.5
		N2-H	6	n	168.1	84.0	84.0	0.0	54.2	29.1	29.1	0.0	-9.8	0.5	9.8	0.5
		N6-H	6	n	83.0	47.4	35.6	-11.9	40.8	19.1	14.2	4.9	-9.8	0.0	4.2	-0.1
	N7	N1-H	6	n	-85.4	-39.2	-46.1	-6.9	-3.4	-16.5	-19.7	3.2	-1.1	-16.7	1.0	-19.7
		N2-H	6	n	183.5	91.8	91.8	0.0	58.5	33.1	33.1	0.0	-9.8	0.5	9.8	0.5
		N7-H	6	n	91.1	41.9	49.2	7.4	41.4	15.2	19.4	-4.2	-9.8	0.1	9.8	0.1
	N8	N1-H	6	n	-100.2	-46.8	-53.5	-6.7	-7.0	-19.4	-22.6	3.2	-1.1	-19.5	1.0	-22.6
		N2-H	6	n	179.0	89.5	89.5	0.0	57.0	31.6	31.6	0.0	-9.8	0.6	9.8	0.6
		N8-H	6	n	76.6	28.6	48.0	19.4	39.0	10.7	19.6	-8.9	-3.4	-0.3	9.8	0.0
	N9	N1-H	6	n	-86.8	-40.7	-46.1	-5.4	-3.0	-17.0	-19.4	2.4	-1.1	-17.3	1.1	-19.4
		N2-H	6	n	190.0	95.0	95.0	0.0	60.1	34.1	34.1	0.0	-9.8	0.5	9.8	0.5
		N9-H	6	n	158.0	79.0	79.0	0.0	57.4	30.0	30.0	0.0	-9.8	0.3	9.8	0.3
	N6	N1-H	7	n	55.3	27.1	28.2	1.1	33.8	15.8	14.9	0.9	-3.5	-1.7	3.3	-1.2
		N2-H	7	n	601.4	300.7	300.7	0.0	171.1	123.0	123.0	0.0	-9.8	0.6	9.8	0.6
		N6-H	7	n	160.5	79.8	80.7	0.9	56.9	32.8	34.3	-1.5	-9.8	0.0	9.8	0.1
	N7	N1-H	7	n	81.0	40.7	40.2	-0.5	40.9	21.0	19.6	1.3	-4.1	-1.1	4.0	-0.7
		N2-H	7	n	595.0	297.5	297.5	0.0	170.2	121.6	121.6	0.0	-9.8	0.6	9.8	0.6
		N7-H	7	n	209.3	104.1	105.2	1.1	71.6	43.4	43.3	0.1	-9.8	0.1	9.8	0.1
	N8	N1-H	7	n	58.9	29.3	29.5	0.2	35.2	16.7	15.3	1.3	-3.6	-1.6	3.4	-1.2
		N2-H	7	n	619.2	309.6	309.6	0.0	176.3	126.5	126.5	0.0	-9.8	0.7	9.8	0.7
		N8-H	7	n	154.7	78.8	75.9	-2.8	55.4	33.6	30.8	2.8	-9.8	0.1	9.8	0.0
	N9	N1-H	7	n	68.1	34.3	33.8	-0.5	37.1	18.6	17.0	1.6	-3.8	-1.3	3.7	-0.9
		N2-H	7	n	599.1	299.5	299.5	0.0	170.5	122.3	122.3	0.0	-9.8	0.7	9.8	0.7
		N9-H	7	n	330.3	165.2	165.2	0.0	101.9	67.5	67.5	0.0	-9.8	0.3	9.8	0.3

Table S13. Different NICS aromaticity indices of ring in pyrido[1,3]diazepines in the water phase. DT – diazepine type, PC - pyridine condensation type, T – tautomer type, R -ring type, P – planarity (p- planar; n- non-planar), I – integral NICS (INICS. ppm/Å), I- – integral NICS for the distances below the plane (ppm/Å), I+ – integral NICS for the distances below the plane (ppm/Å), ΔI=I(-) – I(+)(ppm/Å); NICS(0), NICS(1), NICS(-1), and ΔNICS(-1,1)= NICS(-1) - NICS(1) (ppm). MIN1 and MIN2 are minima of the NICS function for distances below and above the ring plane, respectively, and NICS(MIN1) and NICS(MIN2) are the NICS function values in MIN1 and MIN2.

Water Phase																
DT	PC	T	R	P	I	I-	I+	ΔI	NICS (0)	NICS (-1)	NICS (1)	ΔNICS (-1,1)	MIN1	NICS (MIN1)	MIN2	NICS (MIN2)
pyrido[1,3]diazepines	N6	N1-H	6	n	-85.0	-46.0	-39.0	7.0	-3.0	-19.9	-16.7	-3.1	-1.0	-19.9	1.1	-17.0
		N3-H	6	n	-50.9	-22.6	-28.3	-5.7	4.8	-10.8	-13.0	2.2	-1.2	-11.6	1.2	-13.6
		N6-H	6	p	120.8	63.1	57.7	-5.5	48.5	23.4	21.9	1.5	-9.8	0.2	9.8	0.1
	N7	N1-H	6	n	-78.4	-42.9	-35.6	7.3	-1.9	-18.5	-15.3	-3.2	-1.1	-18.6	1.1	-15.6
		N3-H	6	p	-31.4	-15.7	-15.7	0.0	9.0	-8.0	-8.0	0.0	-1.3	-9.4	1.3	-9.4
		N7-H	6	p	106.0	55.6	50.5	-5.1	44.5	20.6	17.9	2.7	-9.8	0.2	9.8	0.1
	N8	N1-H	6	n	-85.3	-46.5	-38.9	7.6	-3.6	-20.0	-16.6	-3.4	-1.0	-20.0	1.1	-16.9
		N3-H	6	p	-38.3	-17.1	-21.1	-4.0	7.5	-8.5	-10.1	1.6	-1.3	-9.8	1.3	-11.2
		N8-H	6	p	129.1	69.9	59.2	-10.7	50.3	25.6	21.8	3.8	-9.8	0.2	9.8	0.2
	N9	N1-H	6	n	-74.8	-34.6	-40.2	-5.7	-0.3	-15.0	-17.3	2.3	-1.1	-15.3	1.1	-17.5
		N3-H	6	n	-73.0	-33.1	-39.8	-6.7	-0.2	-14.7	-17.2	2.5	-1.1	-15.0	1.1	-17.4
		N9-H	6	p	278.3	139.1	139.1	0.0	89.9	57.7	57.7	0.0	-9.8	0.2	9.8	0.2
	N6	N1-H	7	n	94.6	46.5	48.1	1.6	44.5	22.0	24.2	-2.2	-4.6	-0.5	4.3	-0.9
		N3-H	7	n	176.8	88.1	88.7	0.6	65.8	39.5	39.4	0.1	-7.4	-0.1	8.3	-0.1
		N6-H	7	p	280.9	143.1	137.8	-5.4	89.8	59.2	56.8	2.4	-9.8	0.2	9.8	0.2
	N7	N1-H	7	n	107.6	52.7	54.9	2.2	48.4	24.5	26.7	-2.3	-4.9	-0.4	4.7	-0.7
		N3-H	7	p	214.0	107.0	107.0	0.0	76.0	46.8	46.8	0.0	-9.8	0.0	9.8	0.0
		N7-H	7	p	265.1	133.1	132.0	-1.1	86.1	54.9	54.8	0.0	-9.8	0.2	9.8	0.2
	N8	N1-H	7	n	99.0	48.4	50.7	2.3	46.3	22.7	25.2	-2.5	-4.6	-0.5	4.4	-0.8
		N3-H	7	p	204.0	101.7	102.3	0.6	73.4	44.7	44.9	-0.2	-9.5	0.0	9.8	0.0
		N8-H	7	p	293.0	149.7	143.4	-6.3	92.7	61.4	58.8	2.6	-9.8	0.3	9.8	0.2
	N9	N1-H	7	n	102.5	52.6	49.9	-2.7	46.6	25.9	23.2	2.7	-4.6	-0.7	4.8	-0.4
		N3-H	7	n	128.1	63.7	64.4	0.7	53.4	30.0	29.6	0.5	-5.3	-0.5	5.9	-0.2
		N9-H	7	p	114.4	57.2	57.2	-0.0	47.0	21.3	21.3	0.0	-9.8	0.2	9.8	0.2

Table S14. Different NICS aromaticity indices of ring in pyrido[1,4]diazepines in the water phase. DT – diazepine type, PC - pyridine condensation type, T – tautomer type, R -ring type, P – planarity (p- planar; n- non-planar), I – integral NICS (INICS. ppm/Å), I- – integral NICS for the distances below the plane (ppm/Å), I+ – integral NICS for the distances below the plane (ppm/Å), ΔI=I(-) – I(+)(ppm/Å); NICS(0), NICS(1), NICS(-1), and ΔNICS(-1,1)= NICS(-1) - NICS(1) (ppm). MIN1 and MIN2 are minima of the NICS function for distances below and above the ring plane, respectively, and NICS(MIN1) and NICS(MIN2) are the NICS function values in MIN1 and MIN2.

Water Phase																
DT	PC	T	R	P	I	I-	I+	ΔI	NICS (0)	NICS (-1)	NICS (1)	ΔNICS (-1,1)	MIN1	NICS (MIN1)	MIN2	NICS (MIN2)
pyrido[1,4]diazepines	N6	N1-H	6	n	-71.0	-31.5	-39.5	-3.7	-0.4	-14.1	-17.8	3.7	-1.2	-14.5	1.1	-17.8
		N4-H	6	n	356.1	174.1	182.0	3.7	109.1	71.8	75.5	-3.7	-9.8	0.3	9.8	0.3
		N6-H	6	n	242.4	124.8	117.6	-1.9	77.4	46.6	44.8	1.9	-9.8	0.5	9.8	0.4
	N7	N1-H	6	n	-43.1	-17.7	-25.4	-3.5	6.1	-8.5	-12.0	3.5	-1.3	-9.8	1.2	-12.7
		N4-H	6	p	360.4	180.2	180.2	0.0	99.3	66.8	66.8	0.0	-9.8	1.0	9.8	1.0
		N7-H	6	n	136.5	64.9	71.5	3.5	52.0	23.5	26.9	-3.5	-9.8	0.2	9.8	0.2
	N8	N1-H	6	n	-71.4	-31.1	-40.3	-4.3	-1.1	-13.8	-18.1	4.3	-1.2	-14.2	1.1	-18.1
		N4-H	6	n	118.8	61.5	57.3	-2.7	45.4	23.5	20.8	2.7	-9.8	0.2	9.8	0.2
		N4-H	6	p	241.3	120.7	120.7	0.0	71.6	43.5	43.5	0.0	-9.8	0.7	9.8	0.7
		N8-H	6	p	302.3	151.2	151.2	0.0	90.6	55.7	55.7	0.0	-9.8	0.7	9.8	0.7
	N9	N1-H	6	n	-55.1	-24.0	-31.0	-3.2	4.0	-10.8	-13.9	3.2	-1.2	-11.7	1.2	-14.3
		N4-H	6	n	410.1	201.7	208.5	3.4	122.7	82.6	86.0	-3.4	-9.8	0.4	9.8	0.4
		N4-H	6	p	340.1	170.0	170.0	0.0	94.8	62.7	62.7	0.0	-9.8	1.0	9.8	1.0
		N9-H	6	p	161.2	80.6	80.6	0.0	58.3	30.6	30.6	0.0	-9.8	0.3	9.8	0.3
	N6	N1-H	7	n	134.4	66.5	67.9	-0.9	54.7	31.3	30.5	0.9	-5.3	-0.4	6.8	-0.1
		N4-H	7	n	114.0	59.5	54.5	-3.1	44.6	22.6	19.5	3.1	-9.8	0.2	9.8	0.2
		N6-H	7	n	466.9	235.7	231.3	-1.0	135.3	94.6	93.6	1.0	-9.8	0.6	9.8	0.5
	N7	N1-H	7	n	177.4	88.1	89.3	-0.3	66.0	39.4	39.1	0.3	-7.4	-0.1	9.8	0.0
		N4-H	7	p	895.8	447.9	447.9	0.0	245.2	180.9	180.9	0.0	-9.8	1.2	9.8	1.2
		N7-H	7	n	307.7	155.5	152.2	-2.4	95.9	64.4	62.0	2.4	-9.8	0.3	9.8	0.3
	N8	N1-H	7	n	137.8	68.5	69.3	-1.1	56.2	32.1	31.0	1.1	-5.3	-0.4	6.8	-0.1
		N4-H	7	n	376.2	184.7	191.5	3.2	115.0	76.1	79.3	-3.2	-9.8	0.3	9.8	0.3
		N4-H	7	p	735.0	367.5	367.5	0.0	205.8	149.3	149.3	0.0	-9.8	0.9	9.8	0.9
		N8-H	7	p	591.3	295.6	295.6	0.0	165.9	118.6	118.6	0.0	-9.8	0.8	9.8	0.8
	N9	N1-H	7	n	137.3	68.2	69.2	-0.8	55.3	31.8	31.0	0.8	-5.6	-0.3	6.8	-0.1
		N4-H	7	n	157.3	80.8	76.6	-2.8	54.9	31.3	28.6	2.8	-9.8	0.3	9.8	0.3
		N4-H	7	p	852.1	426.1	426.1	0.0	233.8	172.0	172.0	0.0	-9.8	1.1	9.8	1.1
		N9-H	7	p	336.4	168.2	168.2	0.0	103.3	68.9	68.9	0.0	-9.8	0.3	9.8	0.3

Table S15. Different NICS aromaticity indices of ring in pyrido[1,5]diazepines in the water phase. DT – diazepine type, PC - pyridine condensation type, T – tautomer type, R -ring type, P – planarity (p- planar; n- non-planar), I – integral NICS (INICS. ppm/Å), I- – integral NICS for the distances below the plane (ppm/Å), I+ – integral NICS for the distances below the plane (ppm/Å), ΔI=I(-) – I(+)(ppm/Å); NICS(0), NICS(1), NICS(-1), and ΔNICS(-1,1)= NICS(-1) - NICS(1) (ppm). MIN1 and MIN2 are minima of the NICS function for distances below and above the ring plane, respectively, and NICS(MIN1) and NICS(MIN2) are the NICS function values in MIN1 and MIN2.

Water Phase																	
**DT**	**PC**	**T**	**R**	**P**	**I**	**I-**	**I+**	**ΔI**	**NICS (0)**	**NICS (-1)**	**NICS (1)**	**ΔNICS (-1,1)**	**MIN1**	**NICS (MIN1)**	**MIN2**	**NICS (MIN2)**
pyrido[1,5]diazepines	N6	N1-H	6	n	-74.5	-32.7	-41.8	-9.0	-2.1	-14.8	-18.3	3.6	-1.1	-15.0	1.1	-18.4
		N5-H	6	n	-58.3	-24.9	-33.4	-8.6	2.3	-11.6	-15.0	3.3	-1.2	-12.2	1.1	-15.3
		N6-H	6	p	128.1	64.0	64.0	0.0	48.3	23.1	23.1	0.0	-9.8	0.3	9.8	0.3
	N7	N1-H	6	n	-47.9	-19.3	-28.6	-9.3	4.0	-9.6	-13.4	3.8	-1.2	-10.4	1.2	-13.8
		N5-H	6	n	-66.0	-27.6	-38.4	-10.8	-0.7	-12.7	-17.2	4.5	-1.2	-13.1	1.1	-17.3
		N7-H	6	p	143.2	71.6	71.6	0.0	51.2	25.0	25.0	0.0	-9.8	0.3	9.8	0.3
	N8	N1-H	6	n	-66.0	-27.6	-38.4	-10.8	-0.7	-12.7	-17.2	4.5	-1.2	-13.1	1.1	-17.3
		N5-H	6	n	-47.9	-19.3	-28.6	-9.3	4.0	-9.6	-13.4	3.8	-1.2	-10.4	1.2	-13.8
		N8-H	6	p	143.2	71.6	71.6	0.0	51.2	25.0	25.0	0.0	-9.8	0.3	9.8	0.3
	N9	N1-H	6	n	-58.3	-24.9	-33.4	-8.6	2.3	-11.6	-15.0	3.3	-1.2	-12.2	1.1	-15.3
		N5-H	6	n	-74.5	-32.7	-41.8	-9.0	-2.1	-14.8	-18.3	3.6	-1.1	-15.0	1.1	-18.4
		N9-H	6	p	128.1	64.0	64.0	0.0	48.3	23.1	23.1	0.0	-9.8	0.3	9.8	0.3
	N6	N1-H	7	n	136.8	66.9	69.9	3.0	55.7	31.5	31.5	0.0	-5.3	-0.4	6.8	-0.1
		N5-H	7	n	156.1	77.2	78.9	1.7	60.4	35.5	35.1	0.4	-5.9	-0.2	8.0	-0.1
		N6-H	7	p	356.8	178.4	178.4	0.0	109.3	73.6	73.6	0.0	-9.8	0.3	9.8	0.3
	N7	N1-H	7	n	191.1	94.5	96.6	2.1	70.0	42.1	42.3	-0.2	-8.0	-0.1	9.8	0.0
		N5-H	7	n	161.3	79.3	82.0	2.8	62.5	36.2	36.5	-0.2	-6.2	-0.2	8.3	-0.1
		N7-H	7	p	398.9	199.4	199.4	0.0	119.2	81.8	81.8	0.0	-9.8	0.4	9.8	0.4
	N8	N1-H	7	n	161.3	79.3	82.0	2.8	62.5	36.2	36.5	-0.2	-6.2	-0.2	8.3	-0.1
		N5-H	7	n	191.1	94.5	96.6	2.1	70.0	42.1	42.3	-0.2	-8.0	-0.1	9.8	0.0
		N8-H	7	p	398.9	199.4	199.4	0.0	119.2	81.8	81.8	0.0	-9.8	0.4	9.8	0.4
	N9	N1-H	7	n	156.1	77.2	78.9	1.7	60.4	35.5	35.1	0.4	-5.9	-0.2	8.0	-0.1
		N5-H	7	n	136.8	66.9	69.9	3.0	55.7	31.5	31.5	0.0	-5.3	-0.4	6.8	-0.1
		N9-H	7	p	356.8	178.4	178.4	0.0	109.3	73.6	73.6	0.0	-9.8	0.3	9.8	0.3

Table S16. Different NICS aromaticity indices of ring in pyrido[2,3]diazepines in the water phase. DT – diazepine type, PC - pyridine condensation type, T – tautomer type, R -ring type, P – planarity (p- planar; n- non-planar), I – integral NICS (INICS. ppm/Å), I- – integral NICS for the distances below the plane (ppm/Å), I+ – integral NICS for the distances below the plane (ppm/Å), ΔI=I(-) – I(+)(ppm/Å); NICS(0), NICS(1), NICS(-1), and ΔNICS(-1,1)= NICS(-1) - NICS(1) (ppm). MIN1 and MIN2 are minima of the NICS function for distances below and above the ring plane, respectively, and NICS(MIN1) and NICS(MIN2) are the NICS function values in MIN1 and MIN2.

Water Phase																
**DT**	**PC**	**T**	**R**	**P**	**I**	**I-**	**I+**	**ΔI**	**NICS (0)**	**NICS (-1)**	**NICS (1)**	**ΔNICS (-1,1)**	**MIN1**	**NICS (MIN1)**	**MIN2**	**NICS (MIN2)**
**pyrido[2,3]diazepines**	N6	N2-H	6	p	196.1	98.0	98.0	0.0	63.1	35.6	35.6	0.0	-9.8	0.5	9.8	0.5
		N3-H	6	n	-37.7	-22.0	-15.8	6.2	8.7	-10.6	-7.9	-2.7	-1.3	-11.6	1.3	-9.4
		N6-H	6	n	102.6	48.9	53.7	4.8	46.1	19.4	21.3	-1.9	-9.8	0.0	9.8	0.1
	N7	N2-H	6	p	174.8	87.4	87.4	0.0	57.8	31.3	31.3	0.0	-9.8	0.5	9.8	0.5
		N3-H	6	n	-24.0	-14.8	-9.2	5.6	11.4	-8.0	-5.6	-2.4	-1.3	-9.5	1.4	-7.6
		N7-H	6	n	291.4	145.6	145.8	0.3	89.8	53.9	54.0	-0.1	-9.8	0.6	9.8	0.6
	N8	N2-H	6	p	213.4	106.7	106.7	0.0	67.0	39.0	39.0	0.0	-9.8	0.6	9.8	0.6
		N3-H	6	p	174.8	87.4	87.4	0.0	57.8	31.3	31.3	0.0	-9.8	0.5	9.8	0.5
		N8-H	6	n	86.3	39.0	47.2	8.2	41.8	14.9	18.6	-3.7	-9.8	0.0	9.8	0.0
	N9	N2-H	6	p	165.8	82.9	82.9	0.0	55.4	29.2	29.2	0.0	-9.8	0.5	9.8	0.5
		N3-H	6	p	196.1	98.0	98.0	0.0	63.1	35.6	35.6	0.0	-9.8	0.5	9.8	0.5
		N9-H	6	p	241.7	120.9	120.9	0.0	78.4	45.7	45.7	0.0	-9.8	0.5	9.8	0.5
	N6	N2-H	7	p	564.3	282.1	282.1	0.0	161.3	115.1	115.1	0.0	-9.8	0.6	9.8	0.6
		N3-H	7	n	181.8	91.0	90.7	-0.3	66.3	40.1	40.3	-0.3	-9.8	0.0	8.0	-0.1
		N6-H	7	n	168.4	81.7	86.8	5.1	60.2	33.9	35.9	-2.1	-9.8	0.1	9.8	0.1
	N7	N2-H	7	p	559.0	279.5	279.5	0.0	160.9	114.3	114.3	0.0	-9.8	0.6	9.8	0.6
		N3-H	7	n	225.4	113.0	112.4	-0.7	78.1	49.0	48.7	0.3	-9.8	0.0	9.8	0.0
		N7-H	7	n	524.9	262.3	262.6	0.3	149.0	104.9	105.1	-0.1	-9.8	0.7	9.8	0.7
	N8	N2-H	7	p	607.0	303.5	303.5	0.0	172.6	123.6	123.6	0.0	-9.8	0.7	9.8	0.7
		N3-H	7	p	559.0	279.5	279.5	0.0	160.9	114.3	114.3	0.0	-9.8	0.6	9.8	0.6
		N8-H	7	n	156.7	76.4	80.4	4.0	57.7	31.8	33.3	-1.5	-9.8	0.1	9.8	0.1
	N9	N2-H	7	p	545.9	272.9	272.9	0.0	156.9	111.6	111.6	0.0	-9.8	0.6	9.8	0.6
		N3-H	7	p	564.3	282.1	282.1	0.0	161.3	115.1	115.1	0.0	-9.8	0.6	9.8	0.6
		N9-H	7	p	430.7	215.3	215.3	0.0	126.0	86.7	86.7	0.0	-9.8	0.5	9.8	0.5

Table S17. Different NICS aromaticity indices of ring in pyrido[2,4]diazepines in the water phase. DT – diazepine type, PC - pyridine condensation type, T – tautomer type, R -ring type, P – planarity (p- planar; n- non-planar), I – integral NICS (INICS. ppm/Å), I- – integral NICS for the distances below the plane (ppm/Å), I+ – integral NICS for the distances below the plane (ppm/Å), ΔI=I(-) – I(+)(ppm/Å); NICS(0), NICS(1), NICS(-1), and ΔNICS(-1,1)= NICS(-1) - NICS(1) (ppm). MIN1 and MIN2 are minima of the NICS function for distances below and above the ring plane, respectively, and NICS(MIN1) and NICS(MIN2) are the NICS function values in MIN1 and MIN2.

Water Phase																
**DT**	**PC**	**T**	**R**	**P**	**I**	**I-**	**I+**	**ΔI**	**NICS (0)**	**NICS (-1)**	**NICS (1)**	**ΔNICS (-1,1)**	**MIN1**	**NICS (MIN1)**	**MIN2**	**NICS (MIN2)**
**pyrido[2,4]diazepines**	N6	N2-H	6	p	366.5	183.3	183.3	0.0	102.3	67.8	67.8	0.0	-9.8	1.0	9.8	1.0
		N4-H	6	n	266.0	126.8	139.3	12.5	78.6	46.5	51.4	-4.9	-9.8	0.7	9.8	0.7
		N6-H	6	p	258.2	126.9	131.4	4.5	84.2	50.0	51.5	-1.5	-9.8	0.4	9.8	0.4
	N7	N2-H	6	p	365.4	182.7	182.7	0.0	102.3	68.1	68.1	0.0	-9.8	1.0	9.8	1.0
		N4-H	6	p	443.0	221.5	221.5	0.0	120.4	82.8	82.8	0.0	-9.8	1.2	9.8	1.2
		N7-H	6	n	220.4	105.5	114.9	9.4	74.2	40.4	44.7	-4.3	-9.8	0.3	9.8	0.4
	N8	N2-H	6	p	443.1	221.5	221.5	0.0	120.5	82.8	82.8	0.0	-9.8	1.2	9.8	1.2
		N4-H	6	p	365.4	182.7	182.7	0.0	102.3	68.1	68.1	0.0	-9.8	1.0	9.8	1.0
		N8-H	6	n	220.4	105.5	114.9	9.4	74.2	40.4	44.7	-4.3	-9.8	0.3	9.8	0.4
	N9	N2-H	6	n	266.0	126.8	139.3	12.5	78.6	46.5	51.4	-4.9	-9.8	0.7	9.8	0.7
		N4-H	6	p	366.6	183.3	183.3	0.0	102.3	67.8	67.8	0.0	-9.8	1.0	9.8	1.0
		N9-H	6	p	258.2	126.9	131.4	4.5	84.2	50.0	51.5	-1.5	-9.8	0.4	9.8	0.4
	N6	N2-H	7	p	863.0	431.5	431.5	0.0	236.7	173.4	173.4	0.0	-9.8	1.1	9.8	1.1
		N4-H	7	n	674.9	336.1	338.9	2.8	189.5	135.9	136.6	-0.7	-9.8	0.8	9.8	0.8
		N6-H	7	p	352.8	174.7	178.0	3.3	105.8	70.3	70.6	-0.3	-9.8	0.4	9.8	0.4
	N7	N2-H	7	p	874.4	437.2	437.2	0.0	240.5	175.9	175.9	0.0	-9.8	1.2	9.8	1.2
		N4-H	7	p	1002.3	501.2	501.2	0.0	272.3	200.8	200.8	0.0	-9.8	1.4	9.8	1.4
		N7-H	7	n	333.7	165.4	168.4	3.0	101.6	66.9	67.0	-0.2	-9.8	0.4	9.8	0.4
	N8	N2-H	7	p	1002.5	501.3	501.3	0.0	272.3	200.8	200.8	0.0	-9.8	1.4	9.8	1.4
		N4-H	7	p	874.4	437.2	437.2	0.0	240.5	175.9	175.9	0.0	-9.8	1.2	9.8	1.2
		N8-H	7	n	333.7	165.4	168.4	3.0	101.6	66.9	67.0	-0.2	-9.8	0.4	9.8	0.4
	N9	N2-H	7	n	674.9	336.1	338.9	2.8	189.5	135.9	136.6	-0.7	-9.8	0.8	9.8	0.8
		N4-H	7	p	862.9	431.5	431.5	0.0	236.7	173.4	173.4	0.0	-9.8	1.1	9.8	1.1
		N9-H	7	p	352.8	174.7	178.0	3.3	105.8	70.3	70.6	-0.3	-9.8	0.4	9.8	0.4
